# High expression of CNOT6L contributes to the negative development of type 2 diabetes

**DOI:** 10.1038/s41598-024-76095-5

**Published:** 2024-10-21

**Authors:** Yuna Zhang, Guihong Liu, Haiyan Ding, Bingge Fan

**Affiliations:** Department of Endocrinology, The Fourth Hospital of Hebei Medical University, Shijiazhuang, 050011 China

**Keywords:** CNOT6L, Type 2 diabetes, Molecular targets, Bioinformatics, Endocrine system and metabolic diseases, Metabolic disorders

## Abstract

Objective: Type 2 diabetes (T2D) is a chronic metabolic disorder characterized by reduced responsiveness of body cells to insulin, leading to elevated blood sugar levels. CNOT6L is involved in glucose metabolism, insulin secretion regulation, pancreatic beta-cell proliferation, and apoptosis. These functions may be closely related to the pathogenesis of T2D. However, the exact molecular mechanisms linking CNOT6L to T2D remain unclear. Therefore, this study aims to elucidate the role of CNOT6L in T2D. Methods: The T2D datasets GSE163980 and GSE26168 profiles were downloaded from the Gene Expression Omnibusdatabase generated by GPL20115 and GPL6883.The R package limma was used to screen differentially expressed genes (DEGs). A weighted gene co-expression network analysis was performed. Construction and analysis of the protein–protein interaction (PPI) network, functional enrichment analysis, gene set enrichment analysis, and comparative toxicogenomics database (CTD) analysis were performed. Target Scan was used to screen miRNAs that regulate central DEGs. The results were verified by reverse transcription quantitative real-time polymerase chain reaction (RT-qPCR), western blotting (WB), and blood glucose measurements in mice. Results: A total of 1951 DEGs were identified. GO and KEGG enrichment analysis revealed that differentially expressed genes were mainly enriched in the insulin signaling pathway, ECM-receptor interaction, and PPAR signaling pathway. Metascape analysis indicated enrichment primarily in the cAMP signaling pathway and enzyme-linked receptor protein signaling pathway. WGCNA analysis yielded 50 intersecting genes. PPI network construction and algorithm identification identified two core genes (CNOT6L and GRIN2B), among which CNOT6L gene was associated with multiple miRNAs. CTD analysis revealed associations of core genes with type 2 diabetes, diabetic complications, dyslipidemia, hyperglycemia, and inflammation. WB and RT-qPCR results showed that in different pathways, CNOT6L protein and mRNA levels were upregulated in type 2 diabetes. Conclusion: CNOT6L is highly expressed in type 2 diabetes mellitus, and can cause diabetes complications, inflammation and other physiological processes by regulating miRNA, PPAR and other related signaling pathways, with poor prognosis. CNOT6L can be used as a potential therapeutic target for type 2 diabetes.

**Supplementary Information:**

The online version contains supplementary material available at 10.1038/s41598-024-76095-5.

## Introduction

Type 2 diabetes mellitus (T2D) is a chronic metabolic disorder prevalent among adults, characterized by insulin resistance and insufficient insulin secretion, resulting in impaired glucose utilization and elevated blood glucose levels^1^. T2D poses a significant global public health challenge, with its prevalence increasing annually, closely intertwined with lifestyle alterations, obesity, unhealthy dietary patterns, and sedentary behavior. Vulnerable populations include older adults, obese individuals, those with a familial history of diabetes, and certain ethnic groups (such as Asians and Africans)^2,3^. Insulin resistance diminishes cellular responsiveness, hindering glucose entry into cells and leading to hyperglycemia. Inadequate insulin secretion fails to meet the body’s glucose processing requirements, often accompanied by lipid metabolism abnormalities like hyperlipidemia and elevated cholesterol levels. Hypertension is more prevalent in T2D patients, amplifying the risk of cardiovascular ailments^4^. Hyperglycemia-induced excessive glucose filtration by the kidneys manifests as polyuria, polydipsia, and polyphagia. Inadequate glucose utilization results in insufficient energy supply, causing fatigue and weakness. Elevated blood glucose levels compromise the immune system and microcirculation, delaying wound healing^5^. T2D’s pathogenesis is multifaceted, encompassing insulin resistance, abnormal insulin secretion, and disrupted lipid metabolism. Uncontrolled T2D precipitates severe complications, including hyperglycemia, hypercholesterolemia, and hypertension, elevating the risk of cardiovascular diseases and stroke^6,7^. Though the exact etiology of T2D remains elusive, genetic predisposition, lifestyle factors, obesity, and physical inactivity are implicated. Comprehensive research on T2D, particularly elucidating its molecular intricacies, is imperative for developing improved prevention and treatment modalities, mitigating complication occurrences, and enhancing patient quality of life.

In recent years, bioinformatics has been widespread application and has emerged as a pivotal tool in medical research. Leveraging bioinformatic allows for the analysis of individual genomic and transcriptomic data, laying the groundwork for personalized medicine by predicting disease risks and informing treatment decisions^8^. Moreover, bioinformatics also facilitates hidden patterns and valuable insights from extensive biological datasets, encompassing genomics, transcriptomics, and proteomics data^9^.

CNOT6L encodes a protein crucial for regulatory functions and is part of the CNOT6L protein family, which plays a key role in mRNA degradation and post-transcriptional regulation, thereby influencing gene expression^10^. While initial research on CNOT6L has primarily focused on its involvement in RNA degradation and gene regulation, there has been a growing interest in its potential role in metabolic regulation and disease. Laakso^11^ conducted a comprehensive review on the status and advancements of T2D biomarkers using bioinformatics. Recent large-scale population-based studies and meta-analyses have identified numerous potential genetic and non-genetic biomarkers associated with T2D risk. The integration of genetic variations and physiological characteristics has enhanced the subgroup classification of T2D patients, facilitating the adoption of precision medicine approaches. Some studies have suggested plausible connections between CNOT6L and metabolic disorders, obesity, and insulin sensitivity. However, the precise relationship between CNOT6L and these conditions remains ambiguous and warrants further investigation.

**Fig. 1:**
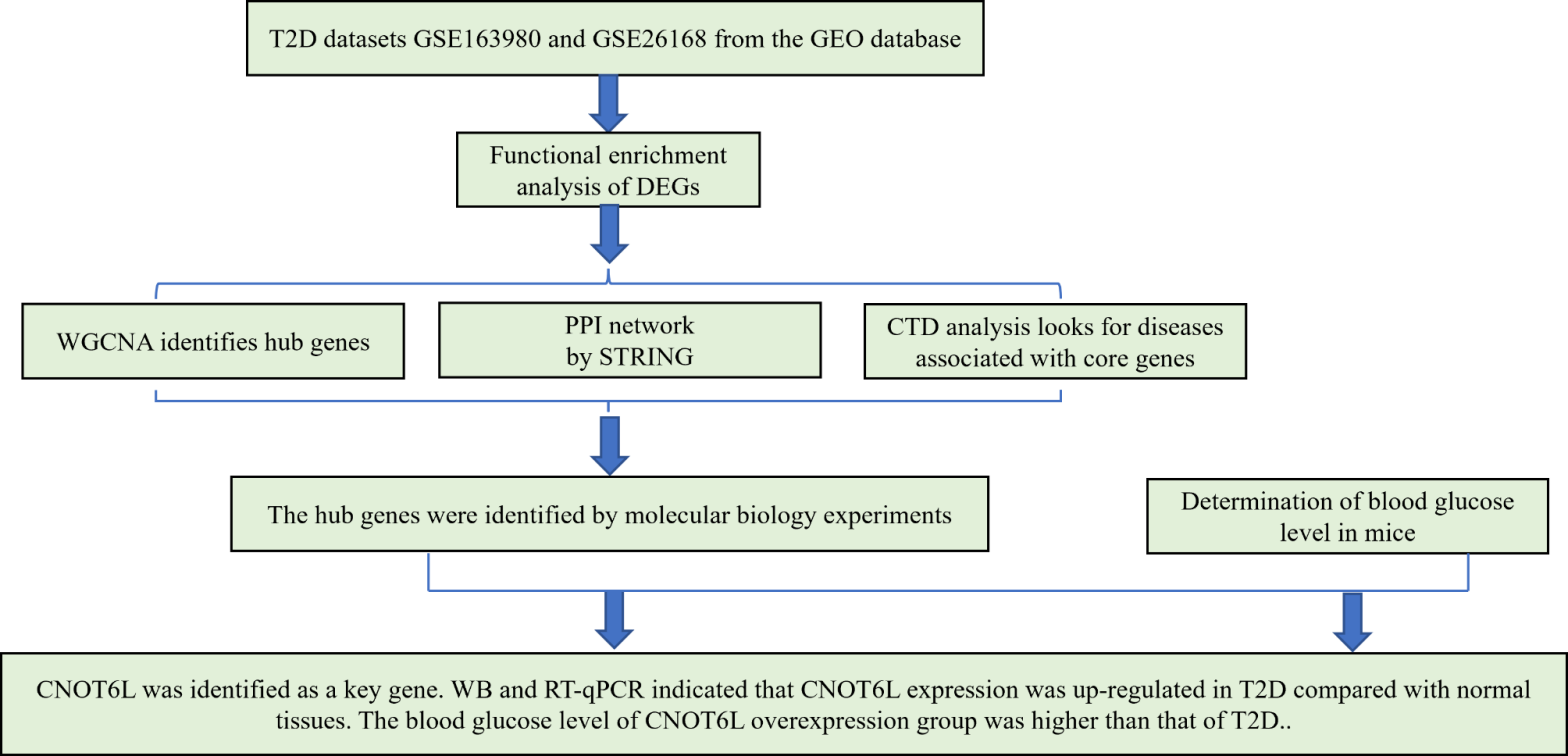
Study flow chart.

Therefore, this study intends to use bioinformatics technology to identify the core genes that distinguish T2D tissues from normal tissues, conduct enrichment and pathway analysis, verify the important role of CNOT6L in T2D using open data sets, and further confirm its influence through basic cell experiments and blood glucose levels in mouse diabetes models. The flow chart is shown in Fig. 1.

## Methods

### Screening the core genes of type 2 diabetes

#### T2D datasets

We accessed T2D datasets GSE163980 and GSE26168 from the Gene Expression Omnibus database (http://www.ncbi.nlm.nih.gov/geo/). These datasets were generated from platforms GPL20115 and GPL6883, respectively. Specifically, GSE163980 comprises data from five T2D cases and five normal whole blood samples, while GSE26168 includes data from nine T2D cases and eight normal whole blood samples. Our objective was to utilize these datasets for the identification of DEGs associated with T2D.

#### Batch effect removal

For the integration and mitigation of batch effects within the GSE163980 and GSE26168 datasets, we initially employed the R package “in Silico Merging.” Following this, the R package “limma” (version 3.42.2) was utilized, along with its Batch Effect removal function, to further refine the merged matrices and alleviate batch discrepancies. This process yielded batch-corrected matrices, ensuring the integrity of subsequent analyses.

#### DEGs identification

The R package limma was utilized to aggregate probes and conduct background correction for the merged matrices obtained from GSE163980 and GSE26168 datasets. The Benjamini–Hochberg method was employed for raw P-value adjustment, while fold changes were calculated using the false discovery rate. Differentially expressed genes (DEGs) were discerned based on a significance threshold of *P* < 0.05, and visualization of the results was facilitated through the creation of a volcano plot.

#### Weighted gene co-expression network analysis (WGCNA)

WGCNA involves several steps. Initially, the median absolute deviation (MAD) was computed for each gene in the merged GSE163980 and GSE26168 matrices, and genes with the lowest 50% MAD were removed. Outlier genes and samples were removed using the good Samples Genes function in the R package WGCNA. A scale-free co-expression network was constructed using the WGCNA package with power parameter selection, adjacency matrix calculation, and topological overlap matrix generation. Hierarchical clustering was performed and the modules were identified. Module preservation analysis and further merging of the similar modules were performed. Using WGCNA analysis, we obtained lists of genes from significant modules to intersect with DEGs, further refining the list of differentially expressed genes. Subsequently, we proceeded with the construction and analysis of the PPI network.

#### Protein–protein interaction (PPI) network construction and analysis

The Search Tool for the Retrieval of Interacting Genes (STRING) database (http://string-db.org/) was used to construct a PPI network of the identified DEGs. Cytoscape was used to visualize and analyze the PPI network. The MCODE algorithm was used to identify significant modules and four algorithms (Matthews correlation coefficient [MCC], maximum neighborhood component [MNC], Radiality, and EcCentricity) were used to identify hub genes.

#### Functional enrichment analysis

Gene Ontology (GO) and Kyoto Encyclopedia of Genes and Genomes (KEGG) analyses (https://www.kegg.jp/kegg/rest/keggapi.html) were performed to assess gene functions and pathways. Cluster Profiler and org. Hs. e.g. db R packages (version 3.1.0) were used to perform enrichment analyses of the DEGs, and the Meta scape database (http://metascape.org/gp/index.html) was used for comprehensive annotation and visualization.

#### Gene set enrichment analysis (GSEA)

GSEA was conducted using the GSEA software to explore enriched pathways and molecular mechanisms in the context of the whole genome. The gene sets were obtained from the Molecular Signatures Database. GSEA was applied to differentially and non-DEGs.

#### Comparative Toxico genomics database (CTD) analysis

The CTD database was used to identify diseases related to core genes and to understand the associations between genes and diseases.

#### miRNA analysis

we utilized online databases such as TargetScan (www.targetscan.org), miRTarBase (mirtarbase.cuhk.edu.cn), and DIANA-TarBase (dianalab.e-ce.uth.gr) to search for miRNA information on target genes. These databases were used to screen for miRNAs regulating core genes.

### Ethics approval

This study was approved by the Ethics Committee of the Fourth Hospital of Hebei Medical University (Ethics batch number 2022KS016). The study protocols was carried out in compliance with the ARRIVE guidelines and in accordance with guide-lines set by the Laboratory Animal Ethical Committee Fourth Hospital Hebei Medical University.

### Experimental approach

We used western blotting and reverse transcription quantitative real-time polymerase chain reaction (RT-qPCR) to detect protein and mRNA expression levels. Plasmids were constructed using the gene knockout and over expressed sequences. Gene knockout and overexpression sequences were placed in the supplementary material.

Animal Model: Mice were divided into control, T2D, T2D with CNOT6L gene over expression(T2D + CNOT6L/OE), and T2D with CNOT6L gene knockout (T2D + CNOT6L/KO) groups.

#### Western blotting (WB)

Western Blotting is a widely used protein analysis technique that involves separating proteins from a sample, transferring them onto a membrane, and detecting target proteins using specific antibodies, allowing for qualitative and quantitative protein analysis. Initially, the sample is lysed and treated with protease inhibitors to obtain protein extracts. The proteins are then separated based on their molecular weight, typically using sodium dodecyl sulfate-polyacrylamide gel electrophoresis (SDS-PAGE). The separated proteins are transferred onto a membrane, commonly polyvinylidene fluoride (PVDF) or nitrocellulose membrane. The membrane is then probed with specific primary antibodies to facilitate specific binding with the target protein. Subsequently, the membrane is washed to remove nonspecifically bound antibodies. Secondary antibodies, typically conjugated with enzymes such as horseradish peroxidase (HRP) or fluorescent dyes, corresponding to the host species of the primary antibodies, are added. Addition of appropriate substrates results in enzyme-mediated luminescent or chromogenic reactions, and signals are captured using an imaging system. The expression levels of target proteins on the membrane are analyzed and quantified by comparing them with those of reference proteins (e.g., GAPDH). For detailed operational steps, refer to the supplementary materials.

#### Real-time polymerase chain reaction (RT-qPCR)

RT-qPCR is a technique used to quantitatively detect the expression levels of specific genes in RNA samples. Firstly, total RNA is extracted from biological samples to ensure the integrity and purity of the RNA. Then, reverse transcriptase is used to transcribe the extracted RNA into corresponding cDNA. Typically, reverse transcription reaction involves the addition of reverse transcriptase, primers, dNTPs (four types of nucleotides), and other reaction components. PCR technology is then employed to amplify the cDNA template generated from reverse transcription using specific primers, while real-time monitoring of the amplification process is achieved through fluorescence dyes. During PCR, the temperature is gradually increased to facilitate DNA denaturation, primer annealing, and DNA polymerase extension, with fluorescence signal changes being recorded. Subsequently, appropriate software is used to analyze the real-time fluorescence data and calculate the relative expression levels of the target gene. For detailed operational steps, refer to the supplementary materials.

#### Detection of blood glucose levels in the mouse model

We used a blood glucose meter (ACCU-CHEK^®^, KunShan, China) to measure the blood glucose levels in ten mice from each of the four groups.

###  Statistical analysis

Molecular expression level data detected by WB and RT-qPCR in different groups, as well as blood glucose level data in mice, were organized using EXCEL. Subsequently, statistical analysis was performed using GraphPad software. After verifying that the data followed a normal distribution, one-way analysis of variance (ANOVA) was conducted to determine the differences in molecular expression and blood glucose levels among different groups. A significance level of *P* < 0.05 was considered statistically significant.

## Results

### Differential Gene expression analysis

**Fig. 2:**
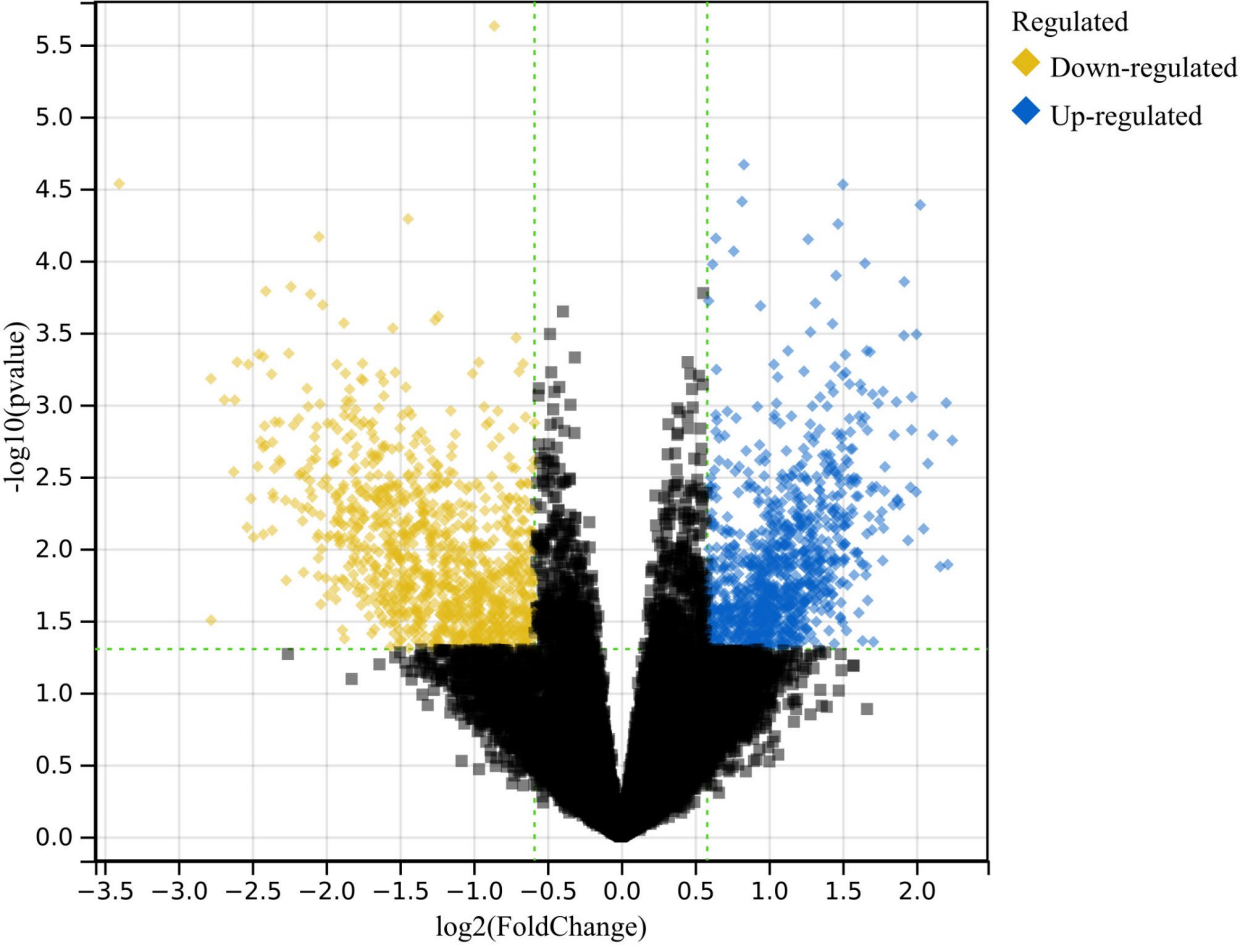
Differential gene analysis. A total of 1951 DEGs.

The merged matrix of type 2 diabetes datasets GSE163980 and GSE26168 identified 1951 differentially expressed genes (DEGs) in total (Fig. 2). Among them, yellow dots represent genes upregulated in T2D relative to normal samples, while blue dots represent downregulated genes. On the y-axis, the smaller the adjusted p-value, the more significant the difference, corresponding to larger -log10 (p-value) values. Therefore, dots in the top left and top right corners represent significantly downregulated and upregulated genes, respectively.

### Functional enrichment analysis

#### DEGs functional enrichment analysis

**Fig. 3:**
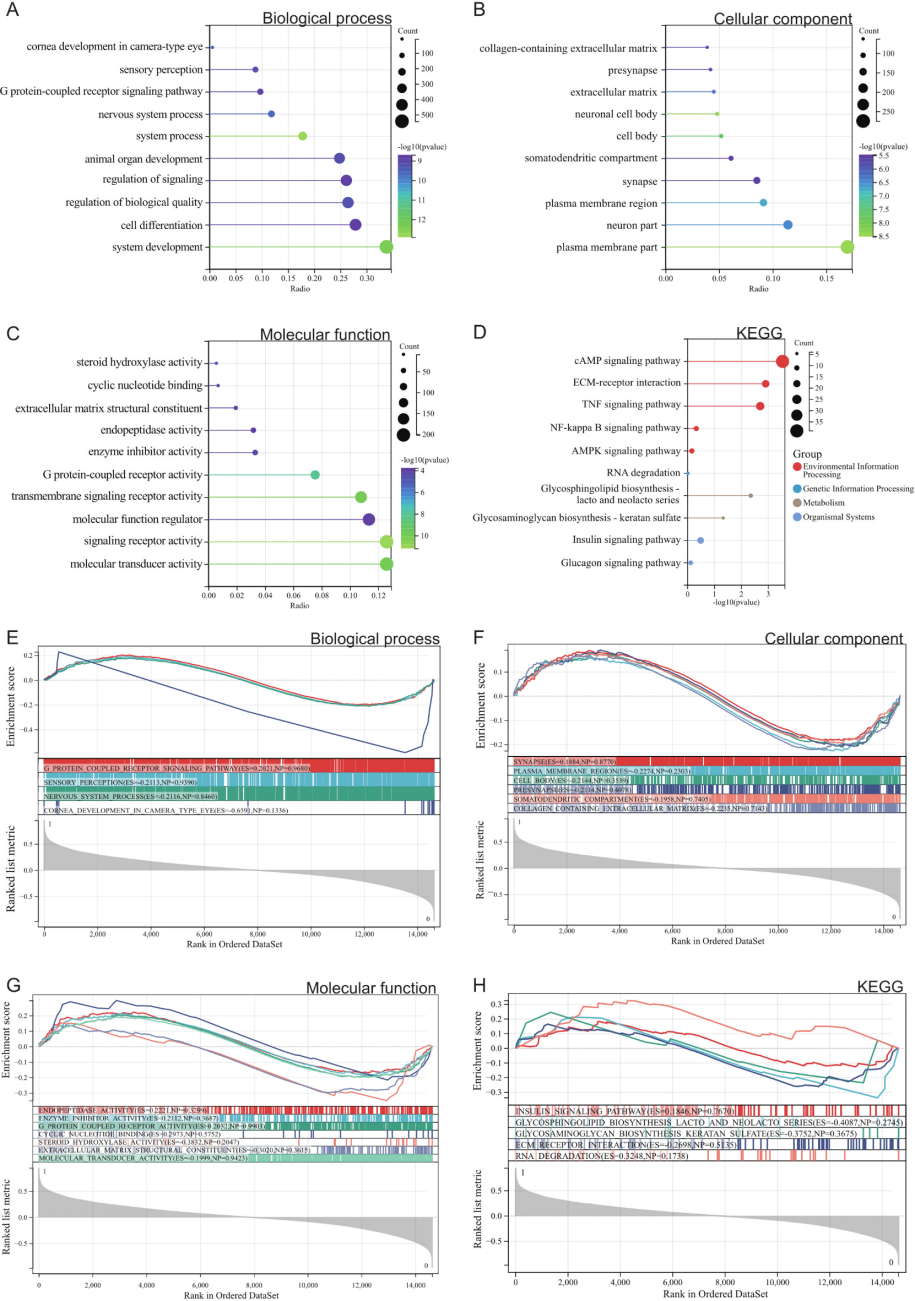
(**A-D**) Results of GOKEGG enrichment analysis of DEGs. (**A**) Biological process analysis. (**B**) Cellular component analysis. (**C**) Molecular function analysis. (**D**) Results of KEGG enrichment analysis. (**E-H**) Results of GSEA enrichment analysis of DEGs. (**E**) Biological process analysis. (**F**) Cellular component analysis. (**G**) Molecular function analysis. (**H**) KEGG enrichment analysis.

We conducted GO and KEGG analyses on these differentially expressed genes. According to the GO analysis, in the Biological Process (BP) enrichment terms, differentially expressed genes are mainly enriched in the G protein-coupled receptor signaling pathway and cell differentiation (Fig. 3A). In the Cellular Component (CC) enrichment terms, they are predominantly enriched in the extracellular matrix containing collagen, extracellular matrix, and membrane fraction (Fig. 3B). In the Molecular Function (MF) enrichment terms, they are mainly enriched in molecular function regulators, transmembrane signaling receptor activity, enzyme inhibitor activity, and G protein-coupled receptor activity (Fig. 3C). In the KEGG analysis, we found that DEGs are primarily concentrated in pathways such as the insulin signaling pathway, cAMP signaling pathway, PPAR signaling pathway, TNF signaling pathway, and ECM-receptor interaction (Fig. 3D). These pathways play crucial roles in the occurrence and progression of type 2 diabetes, thus we will focus on the genes associated with these pathways.

#### GSEA analysis

Furthermore, we performed Gene Set Enrichment Analysis (GSEA) on the entire genome to identify potential enrichments among non-differentially expressed genes and validate the results of differentially expressed genes. The intersection of enrichment terms from GO and KEGG with differentially expressed genes is depicted in the figure, indicating that differentially expressed genes are mainly enriched in pathways such as the insulin signaling pathway, ECM-receptor interaction, and PPAR signaling pathway (Fig. 3E, F, G, H).

####  Metascape enrichment analysis

**Fig. 4:**
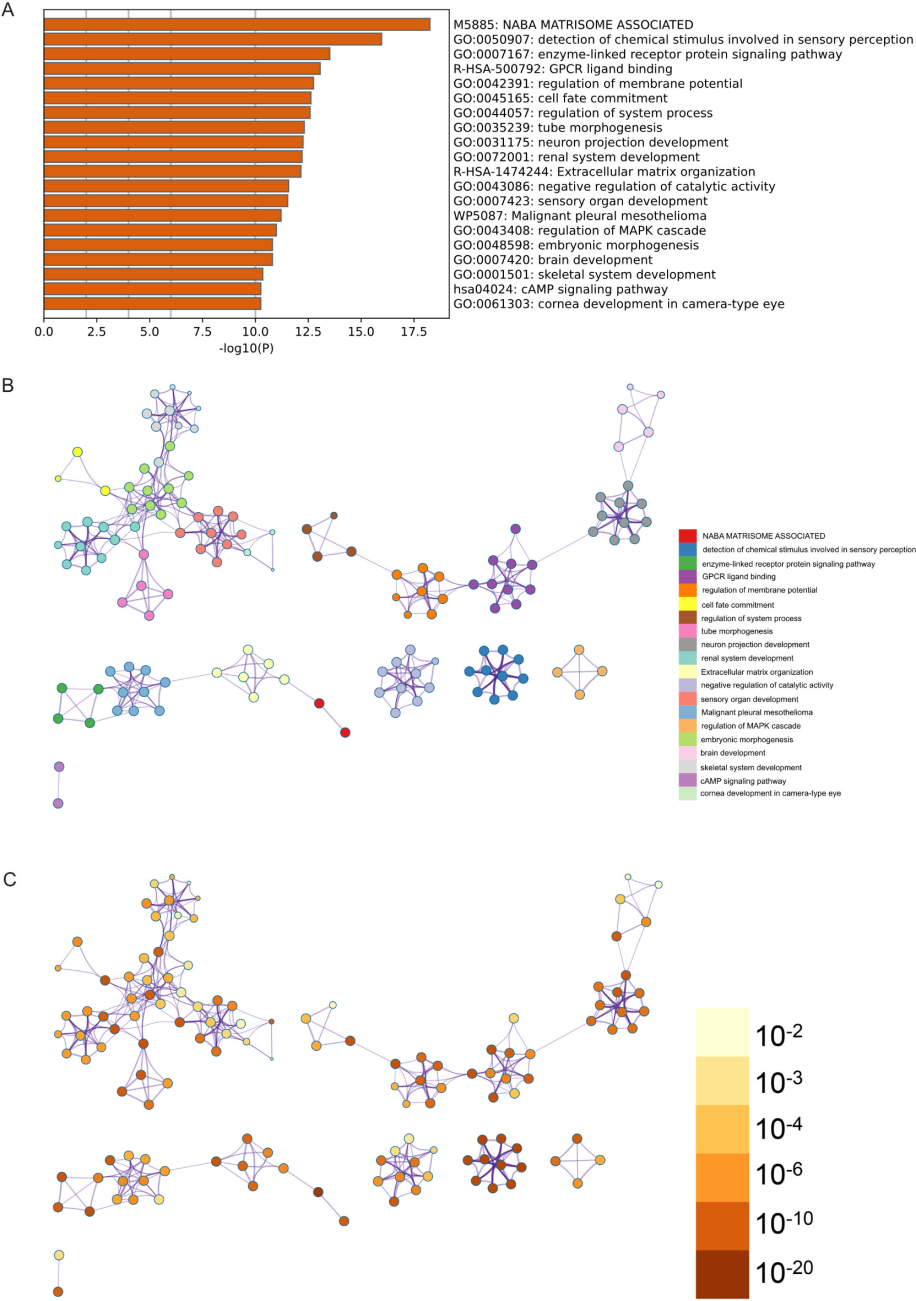
Metascape enrichment analysis. (**A**) Bar graph of enriched terms across input gene lists, colored by p-values. (**B**) Network of enriched terms: colored by cluster ID, where nodes that share the same cluster ID are typically close to each other. (**C**) colored by p-value, where terms containing more genes tend to have a more significant p-value.

In addition, we inputted these lists of differentially expressed genes into the Metascape online analysis tool for supplementary GO and KEGG analysis. In the enrichment items of Metascape, GO enrichment items such as the cAMP signaling pathway and enzyme-linked receptor protein signaling pathway were observed (Fig. 4A). Additionally, we generated enrichment networks colored by enrichment term and p-value to visually represent the associations and confidence levels of each enrichment item (Fig. 4B, C).

### WGCNA

**Fig. 5:**
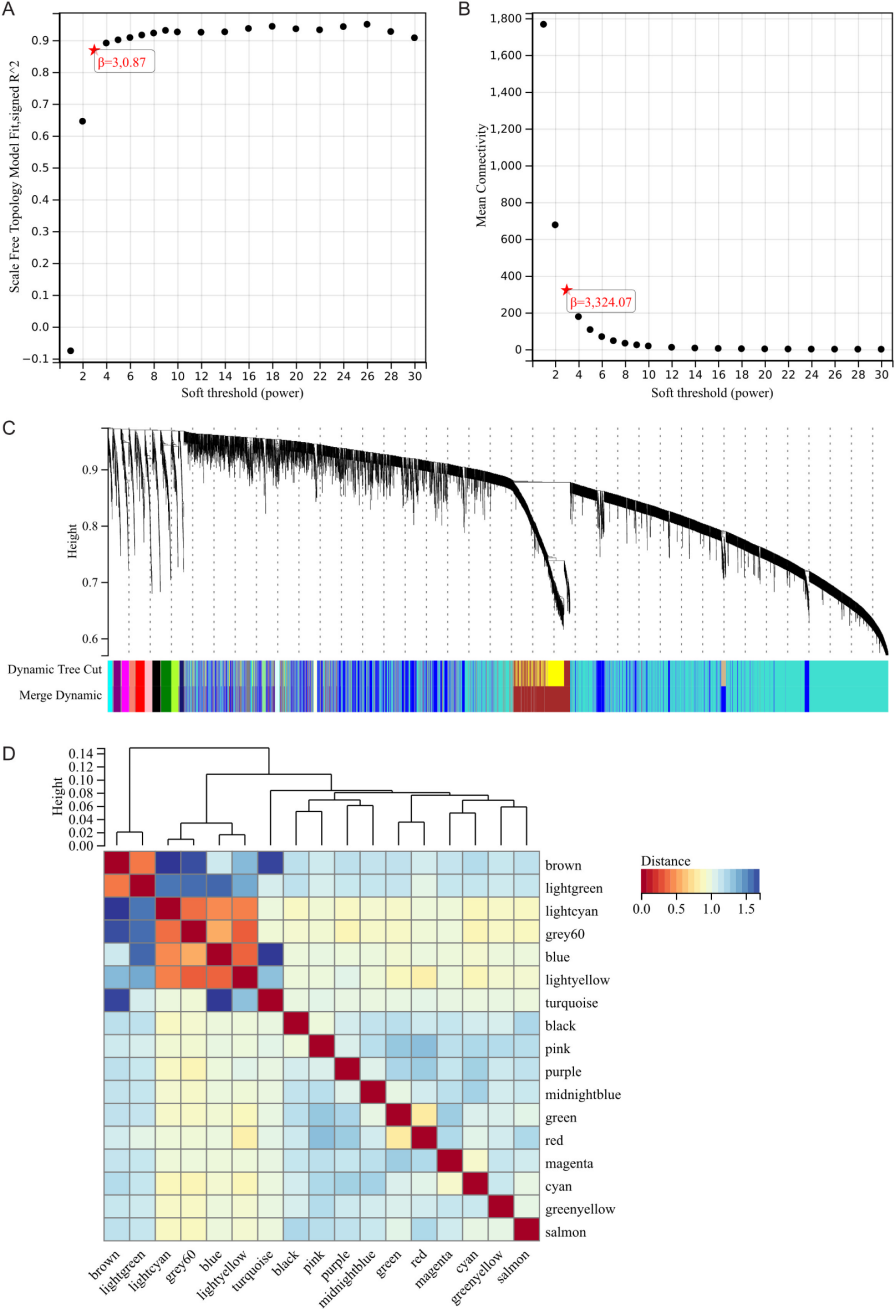
WGCNA analysis. (**A**) β = 3,0.87. β = 3,324.07. **(B, C) **The hierarchical clustering tree of all genes was constructed, and 17 important modules were generated.

**Fig. 6:**
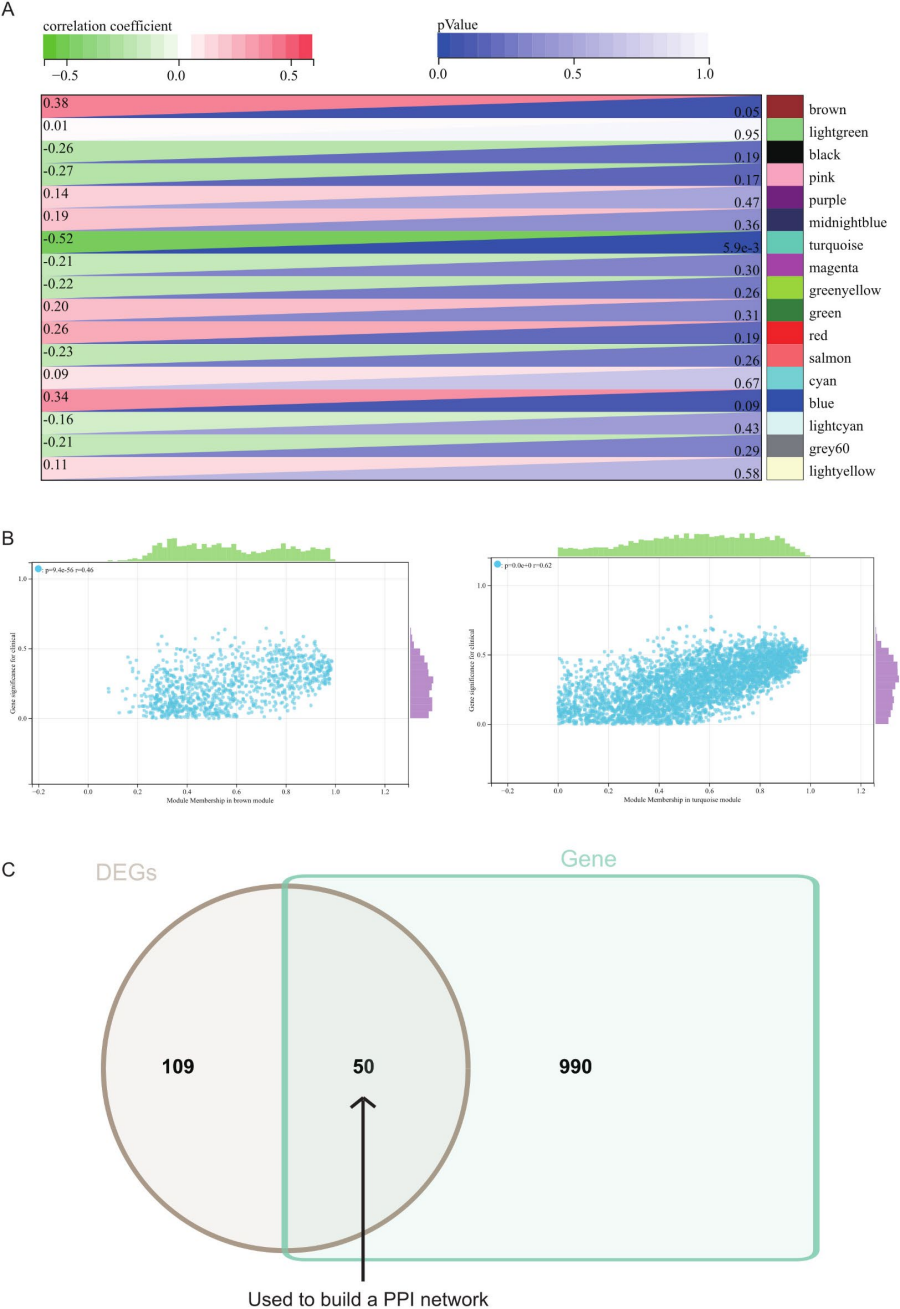
(**A**) The heat map of correlation between modules and phenotypes. (**B**) The scatter map of correlation between GS and MM of related hub genes. (**C**) The DEGs screened by WGCNA and DEGs was used to obtain venn map. 50 intersection genes were obtained.

The selection of soft threshold power is a crucial step in WGCNA analysis. We performed network topology analysis to determine the soft threshold power. In our WGCNA analysis, the soft threshold power was set to 9, which is the lowest power of 0.9 for the scale-free topology fit index (Fig. 5A, B). A hierarchical clustering tree of all genes was constructed, resulting in a total of 17 modules (Fig. 5C). Interactions between significant modules were analyzed (Fig. 5D), and module-trait correlation heatmaps (Fig. 6A) and scatter plots of gene significance (GS) versus module membership (MM) for relevant hub genes were generated (Fig. 6B).

We calculated the correlation between module eigengenes and gene expression to obtain MM. Based on a cutoff criterion (|MM| > 0.8), two highly interconnected genes were identified as hub genes in clinically significant modules. We intersected these hub genes from the two key modules with the relevant genes from the crucial pathways identified in the aforementioned analysis, resulting in 50 intersecting genes (Fig. 6C). These genes may play pivotal roles in type 2 diabetes and were subsequently used for constructing and analyzing the protein-protein interaction (PPI) network to identify target genes.

### Construction and analysis of PPI network

**Fig. 7:**
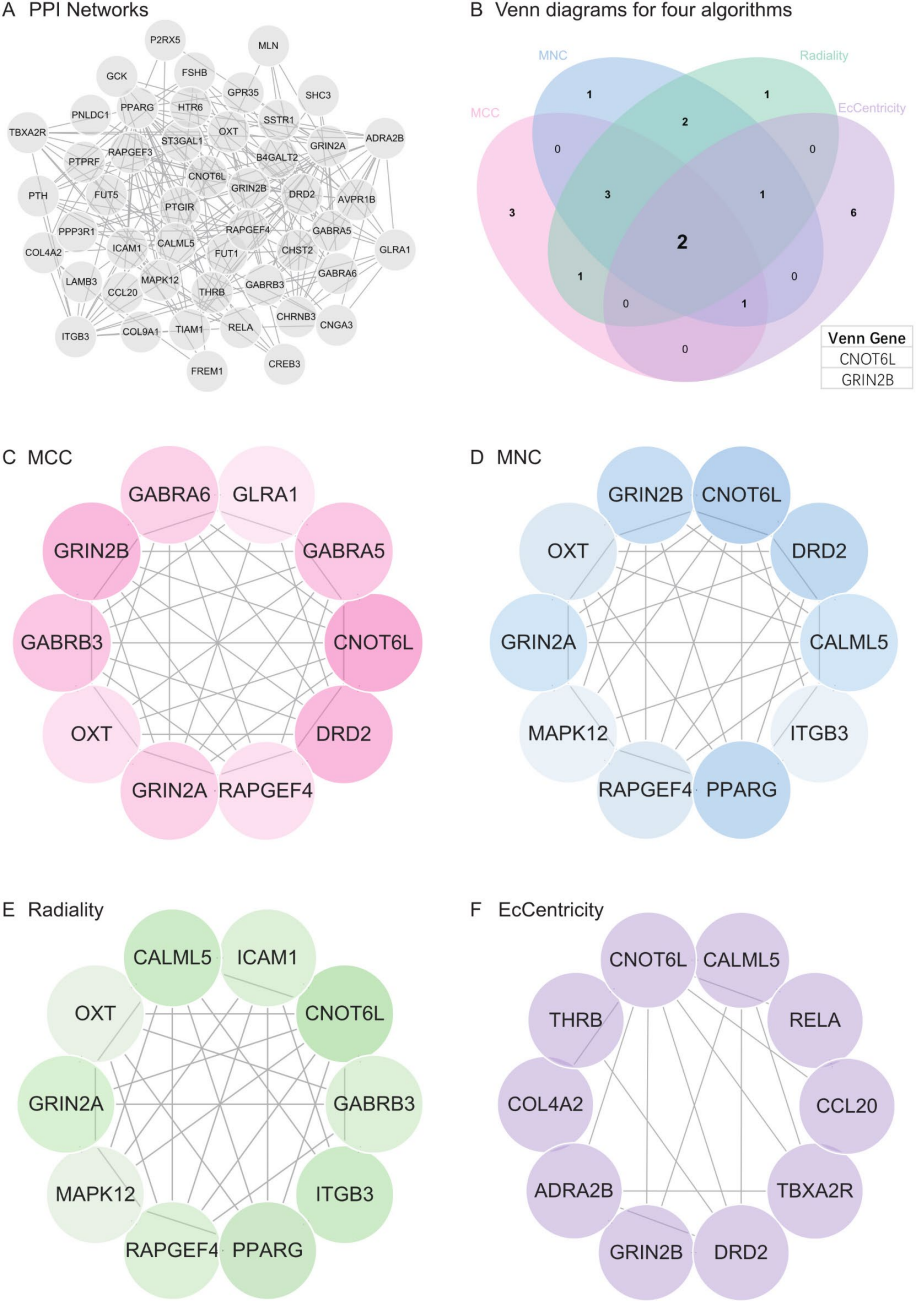
Construction and analysis of protein-protein interaction (PPI) networks. (**A**) Construct the PPI network of DEGs using STRING online database and utilize Cytoscape software for analysis. (**B**) Core genes (CNOT6L, GRIN2B) were obtained by merging using Venn diagrams. (**C**) MCC was used to identify the central gene. (**D**) MNC was used to identify the central gene. (**E**) Radiality was used to identify the central gene. (**F**) EcCentricity was used to identify the central gene.

Using the results from the previous analysis, we constructed a protein-protein interaction (PPI) network for the gene list. The PPI network was built using the STRING online database and analyzed using Cytoscape software (Fig. 7A). Subsequently, four algorithms were employed to identify hub genes, and the Venn diagram was used to obtain the intersection (Fig. 7B). The results of the MCC, MNC, Radiality, and Eccentricity algorithms are depicted in Fig. 7C-F. Finally, we identified two core genes (CNOT6L and GRIN2B).

### Prediction and functional annotation of miRNA associated with hub gene

We inputted the hub gene list into miRNA prediction websites to identify relevant miRNAs, enhancing our understanding of gene expression regulation (Table 1).

**Table 1:** A summary of miRNAs that regulate hub genes.

	Gene	MIRNA		
1	CNOT6L	hsa-miR-9-5p		
2	EHMT2	hsa-miR-1-3p	hsa-miR-206	hsa-miR-613
3	HIST1H2BO	hsa-miR-760	hsa-miR-542-3p	
4	HIST1H4B	None		
5	HIST2H4A	None		
6	HIST1H4F	None		

According to the predictions from the TargetScan website, the relevant miRNA for the CNOT6L gene is hsa-miR-9-5p, and for the GRIN2B gene, they are hsa-miR-204-5p and hsa-miR-211-5p.

Predictions from the miRTarBase website revealed that the relevant miRNAs for the CNOT6L gene are hsa-miR-106b-5p, hsa-miR-186-5p, and hsa-miR-146a-5p, while for the GRIN2B gene, they are hsa-miR-1277-5p, hsa-miR-297, and hsa-miR-3924.

According to predictions from the DIANA-TarBase website, the relevant miRNAs for the CNOT6L gene are hsa-miR-17-5p, hsa-miR-18a-5p, and hsa-miR-19a-3p, while for the GRIN2B gene, they are hsa-miR-107, hsa-miR-15a-5p, and hsa-miR-26a-5p.

### CTD analysis

**Fig. 8:**
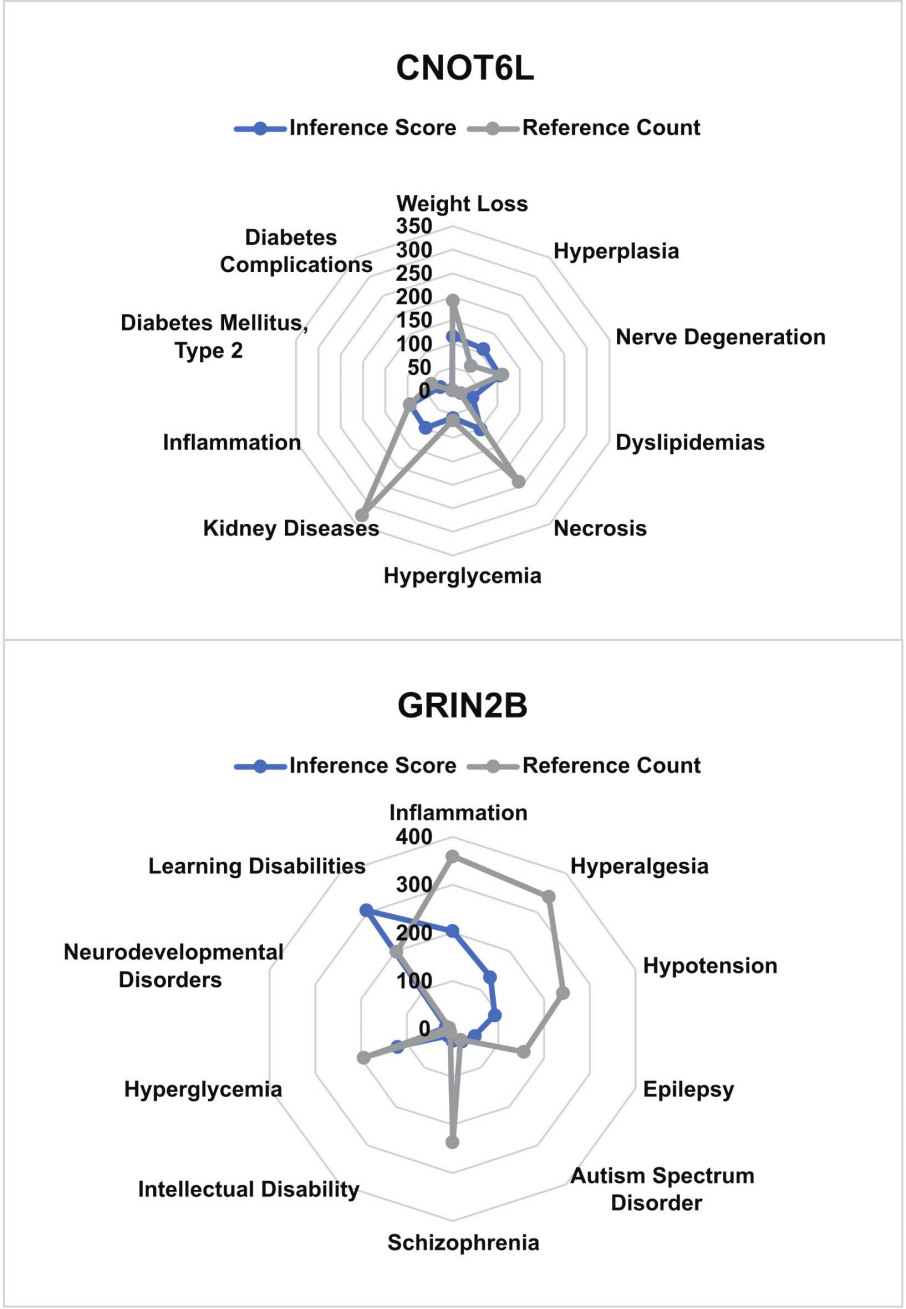
CTD analysis. Core genes (CNOT6L, GRIN2B) are associated with type 2 diabetes, diabetes complications, dyslipidemia, hyperglycemia and inflammation.

We inputted the hub gene list into the CTD website to explore diseases associated with the core genes, enhancing our understanding of gene-disease associations. We found that the core genes (CNOT6L and GRIN2B) are associated with type 2 diabetes, diabetic complications, dyslipidemia, hyperglycemia, and inflammation (Fig. 8). Combining the analyses mentioned above, we believe that the analysis results of the core gene CNOT6L may be intricately linked to type 2 diabetes.

###  Protein and blood glucose levels in mice with type 2 diabetes

#### CNOT6L protein expression

**Fig. 9:**
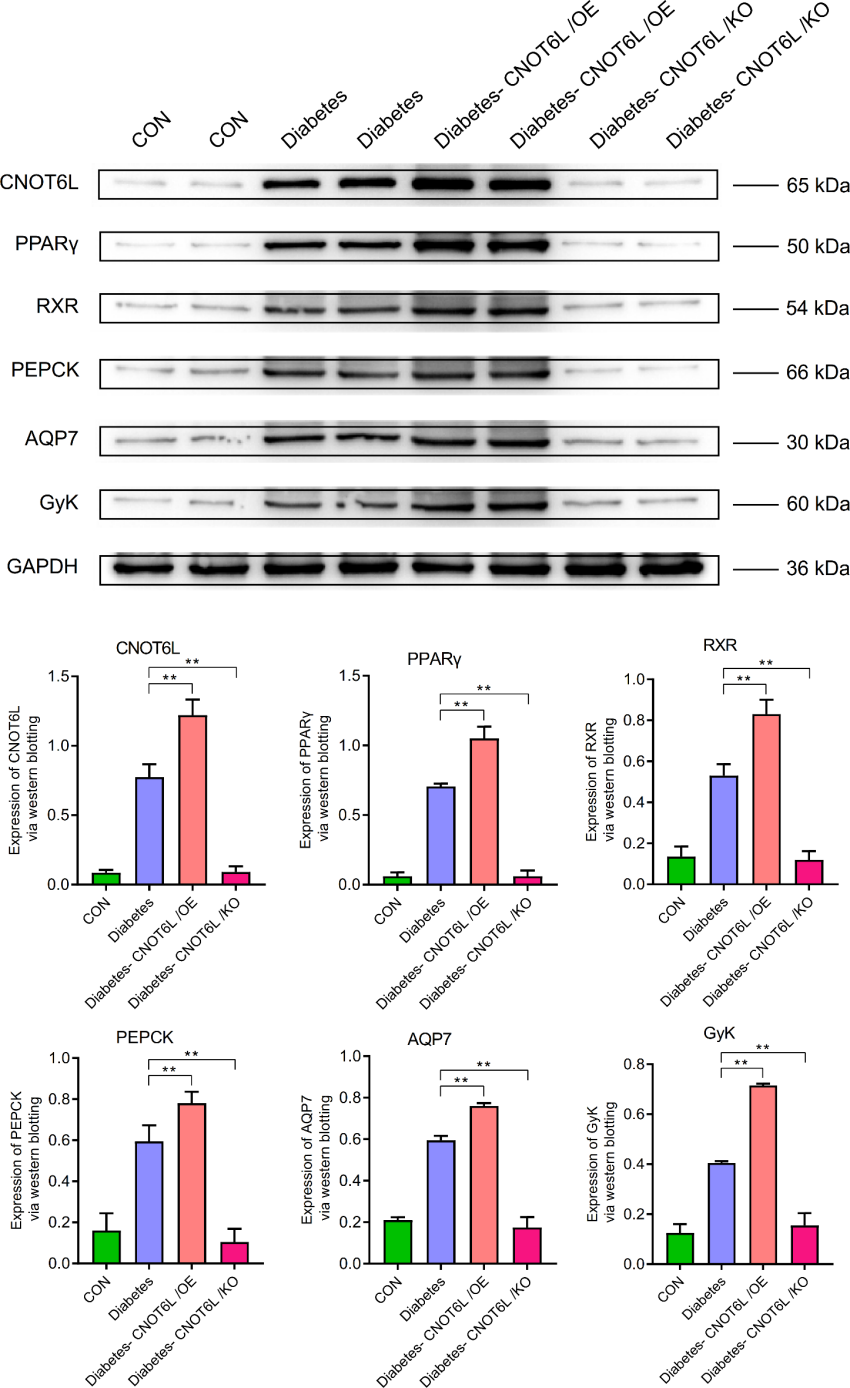
Expression of CNOT6L, PPARγ, RXR, PEPCK, AQP7, and GYK in the blood of type 2 diabetes mellitus mice. Protein expression levels were determined by western blotting. A representative blot comparing control (CON), type 2 diabetes (Diabetes), type 2 diabetes CNOT6L gene overexpression (Diabetes-CNOT6L/OE), and type 2 diabetes CNOT6L gene knockout (Diabetes-CNOT6L/KO) groups is shown, with each sample run in duplicate. GAPDH was used as the internal control. The results are presented as mean ± standard deviation of independent 10 experiments.** *P* < 0.01; *** *P* < 0.001.

WB showed that in the PPAR pathway, the expression levels of CNOT6L, PPARγ, RXR, PEPCK, AQP7, and GYK were higher in the T2D group than in the control group. In the T2D-CNOT6L/OE group, the expression levels were significantly higher than those in the T2D group (*P* < 0.01), whereas in the T2D-CNOT6L/KO group, the expression levels were significantly lower than those in the T2D group (*P* < 0.01) (Fig. 9).

**Fig. 10:**
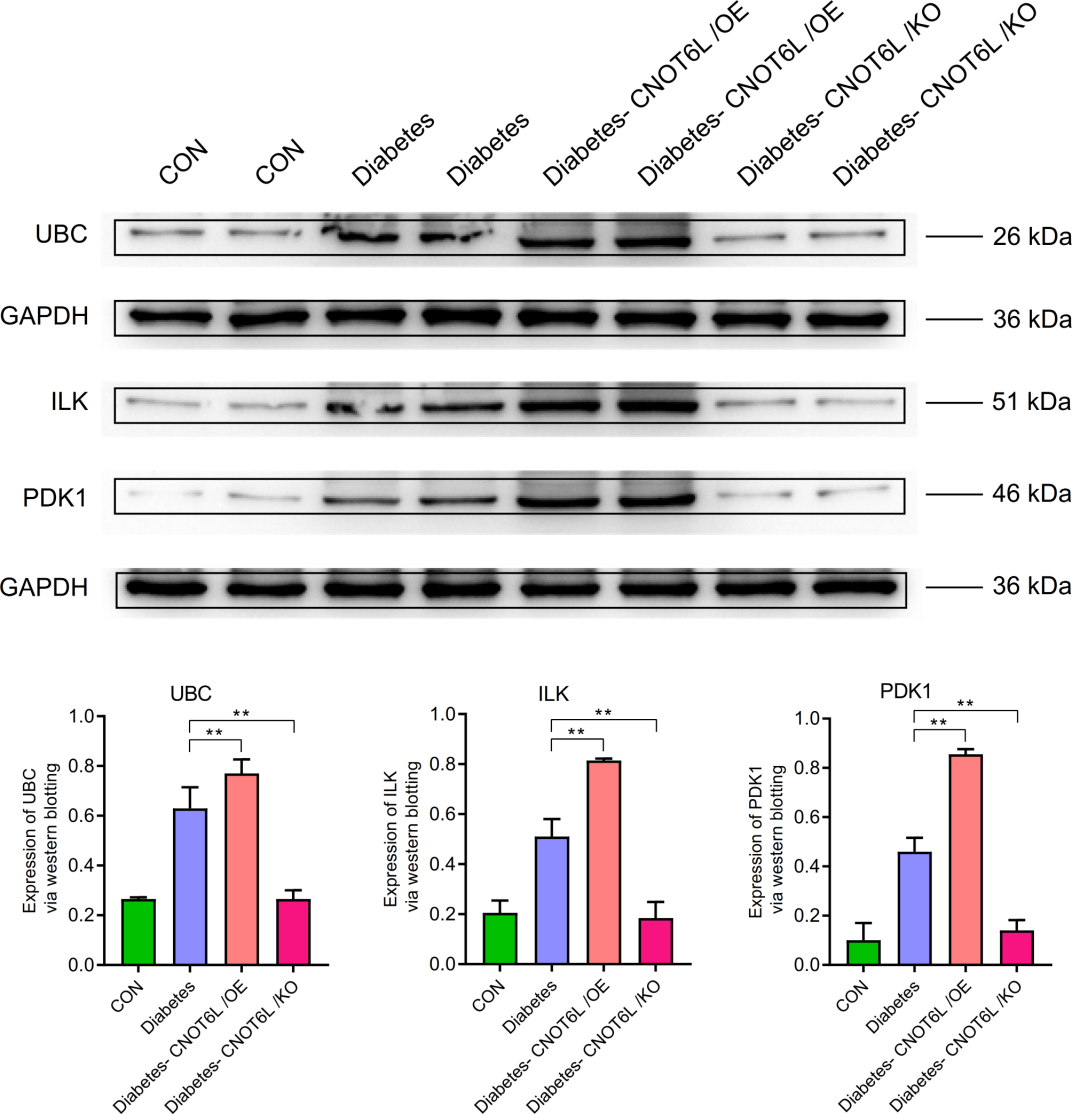
Expression of ubiquitination-related genes UBC, ILK, and PDK1 in the blood of type 2 diabetes mellitus mice. Protein expression levels were determined by western blotting. A representative blot comparing control (CON), type 2 diabetes (Diabetes), type 2 diabetes CNOT6L gene overexpression (Diabetes-CNOT6L/OE), and type 2 diabetes CNOT6L gene knockout (Diabetes-CNOT6L/KO) groups is shown, with each sample run in duplicate. GAPDH was used as the internal control. The results are presented as mean ± standard deviation of independent 10 experiments. ** *P* < 0.01.

In the ubiquitination pathway, the expression levels of UBC, ILK, and PDK1 were higher in the T2Dgroup than those in the control group. In the T2D-CNOT6L/OE group, the expression levels were significantly higher than those in the T2D group (*P* < 0.01), whereas in the T2D-CNOT6L/KO group, the expression levels were significantly lower than those in the T2Dgroup (*P* < 0.01) (Fig. 10).

**Fig. 11:**
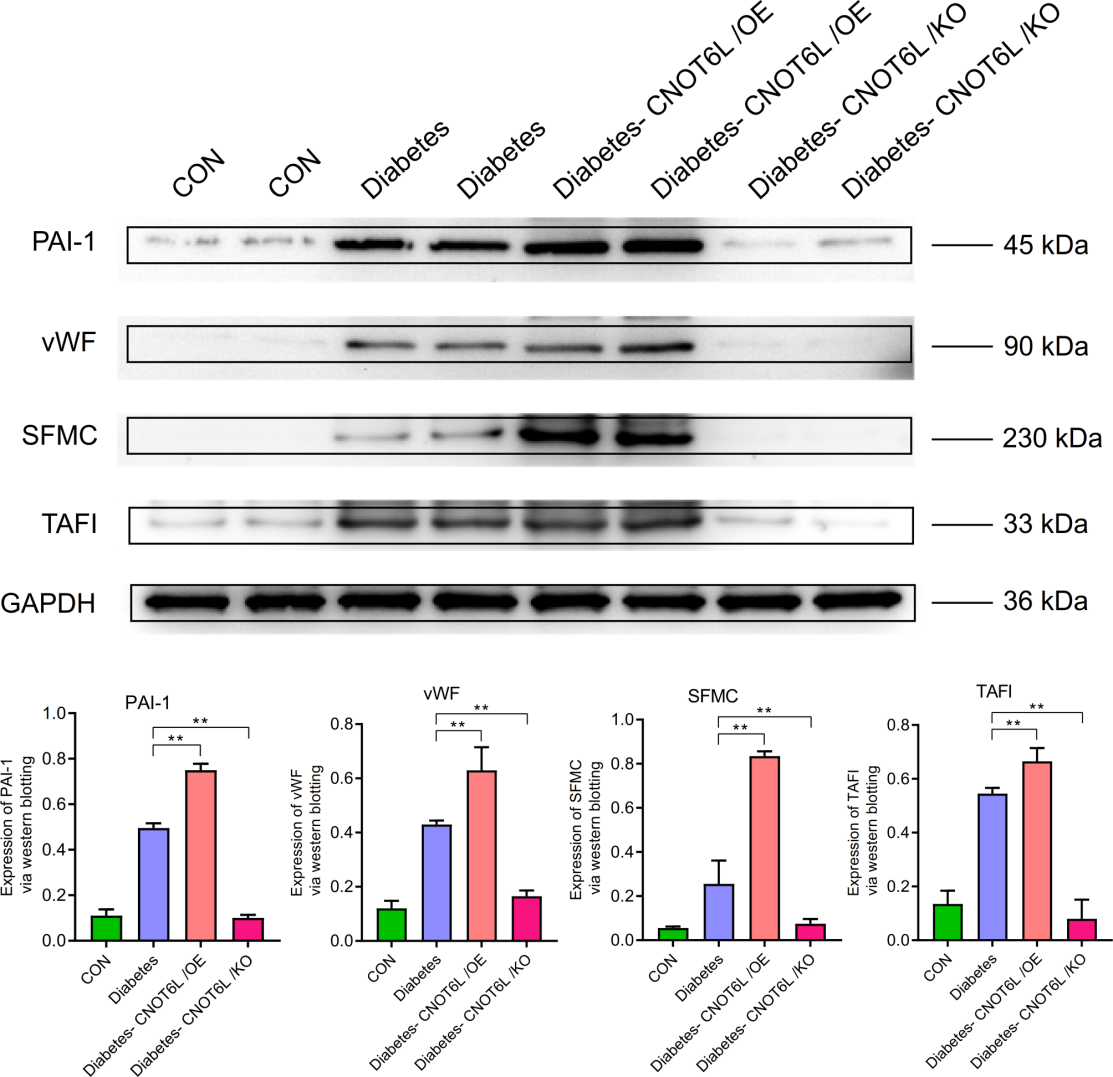
Expression of clotting-related genes PAI-1, vWF, SFMC, and TAFI in the blood of type 2 diabetes mellitus mice. Protein expression levels were determined by western blotting. A representative blot comparing control (CON), type 2 diabetes (Diabetes), type 2 diabetes CNOT6L gene overexpression (Diabetes-CNOT6L/OE), and type 2 diabetes CNOT6L gene knockout (Diabetes-CNOT6L/KO) groups is shown, with each sample run in duplicate. GAPDH was used as the internal control. The results are presented as mean ± standard deviation of independent 10 experiments. ** *P* < 0.01.

**Fig. 12:**
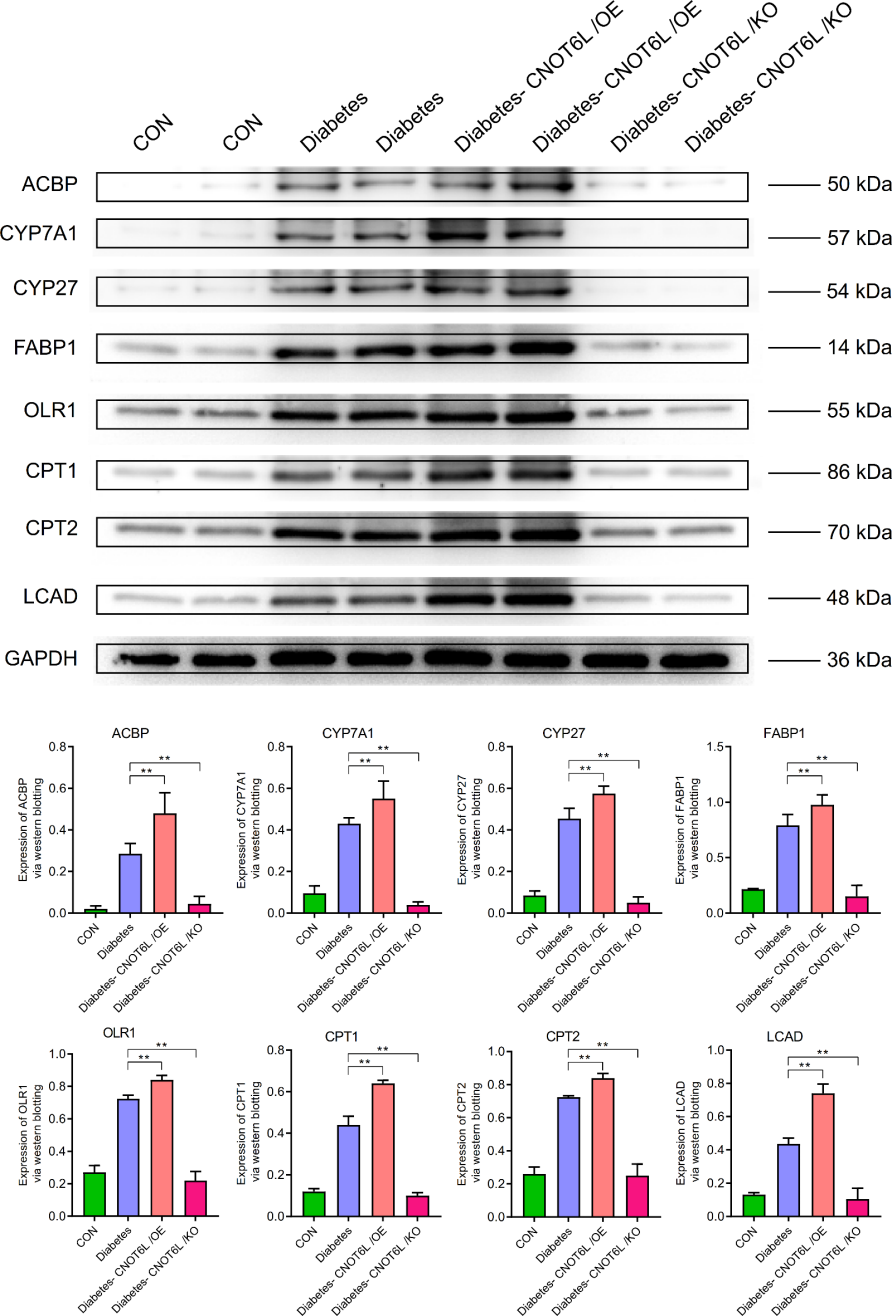
Expression of clotting-related genes ACBP, CYP7A1, CYP27, FABP1, OLR1, CPT1, CPT2, and LCAD in the blood of type 2 diabetes mellitus mice. Protein expression levels were determined by western blotting. A representative blot comparing control (CON), type 2 diabetes (Diabetes), type 2 diabetes CNOT6L gene overexpression (Diabetes-CNOT6L/OE), and type 2 diabetes CNOT6L gene knockout (Diabetes-CNOT6L/KO) groups is shown, with each sample run in duplicate. GAPDH was used as the internal control. The results are presented as mean ± standard deviation of independent 10 experiments. ** *P* < 0.01.

In terms of coagulation, the expression levels of PAI-1, vWF, SFMC, TAFI, ACBP, CYP7A1, CYP27, FABP1, OLR1, CPT1, CPT2, and LCAD were higher in the T2Dgroup than in the control group. In the T2D-CNOT6L/OE group, the expression levels were significantly higher than those in the T2D group (*P* < 0.01), whereas in the T2D-CNOT6L/KO group, the expression levels were significantly lower than those in the T2D group (*P* < 0.01) (Figs. 11 and 12).

**Fig. 13:**
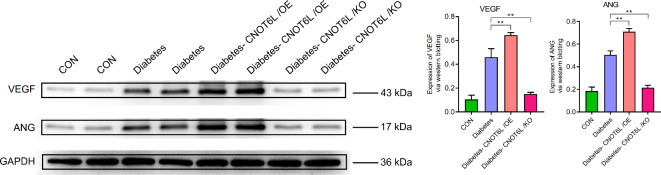
Expression of VEGF and ANG in the blood of type 2 diabetes mellitus mice. Protein expression levels were determined by western blotting. A representative blot comparing control (CON), type 2 diabetes (Diabetes), type 2 diabetes CNOT6L gene overexpression (Diabetes-CNOT6L/OE), and type 2 diabetes CNOT6L gene knockout (Diabetes-CNOT6L/KO) groups is shown, with each sample run in duplicate. GAPDH was used as the internal control. The results are presented as mean ± standard deviation of independent 10 experiments. ** *P* < 0.01.

**Fig. 14:**
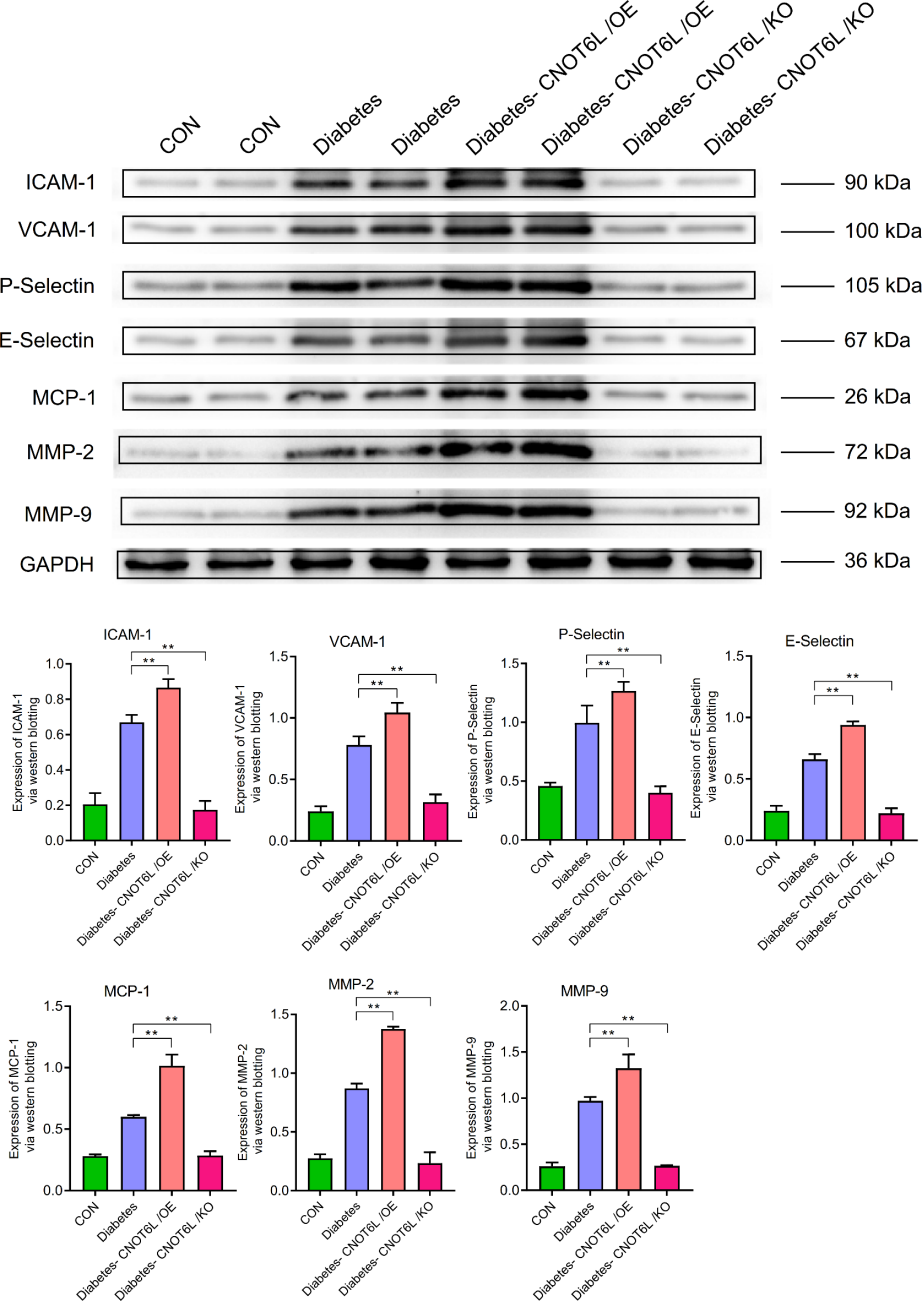
Expression of ICAM-1, VCAM-1, P-selectin, E-selectin, MCP-1, MMP-2, and MMP-9 in the blood of type 2 diabetes mellitus mice. Protein expression levels were determined by western blotting. A representative blot comparing control (CON), type 2 diabetes (Diabetes), type 2 diabetes CNOT6L gene overexpression (Diabetes-CNOT6L/OE), and type 2 diabetes CNOT6L gene knockout (Diabetes-CNOT6L/KO) groups is shown, with each sample run in duplicate. GAPDH was used as the internal control. The results are presented as mean ± standard deviation of independent 10 experiments. ** *P* < 0.01.

In other pathways, the expression levels of VEGF, ANG, ICAM-1, VCAM-1, P-selectin, E-selectin, MCP-1, MMP-2, and MMP-9 were similar to the expression patterns of the aforementioned genes in the different groups (Figs. 13 and 14). See supplementary material for complete strips.

####  mRNA expression of CNOT6L in mice blood cells

**Fig. 15:**
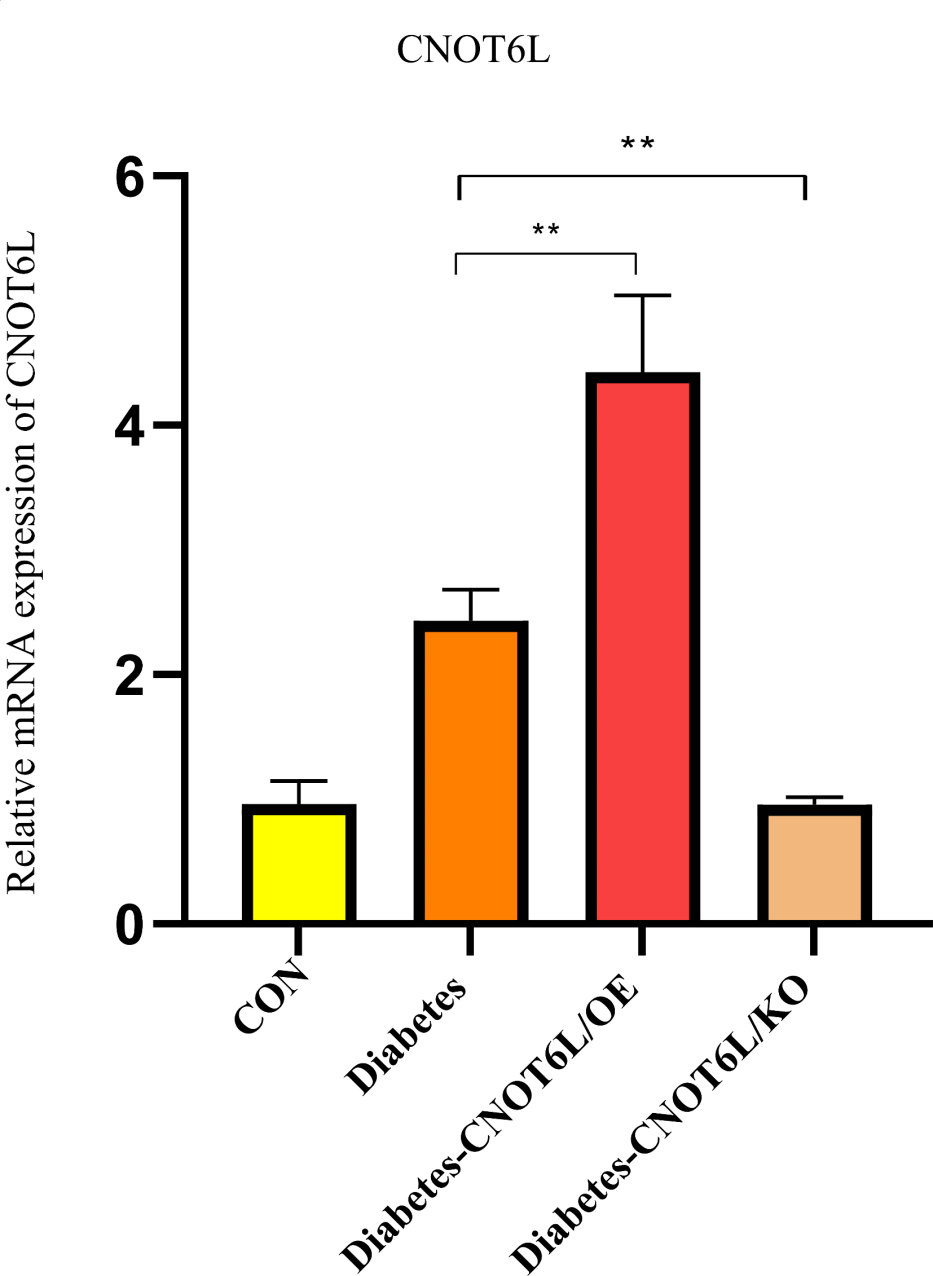
Relative mRNA expression of CNOT6L in the blood of type 2 diabetes mellitus mice. Expression levels were determined by RT-qPCR for the control (CON), type 2 diabetes (Diabetes), type 2 diabetes CNOT6L gene overexpression (Diabetes-CNOT6L/OE), and type 2 diabetes CNOT6L gene knockout (Diabetes-CNOT6L/KO) groups. The results are presented as mean ± standard deviation of independent 10 experiments. ** *P* < 0.01.

RT-qPCR showed that, compared to normal samples (controls), the relative mRNA expression level of CNOT6L was elevated in the T2D group. In the T2D-CNOT6L/OE group, the mRNA expression level of CNOT6L was significantly higher than that in the T2D group (*P* < 0.01), whereas it was decreased in the T2D-CNOT6L/KO group (*P* < 0.01) (Fig. 15).

#### Blood glucose levels in diabetic mice

**Fig. 16:**
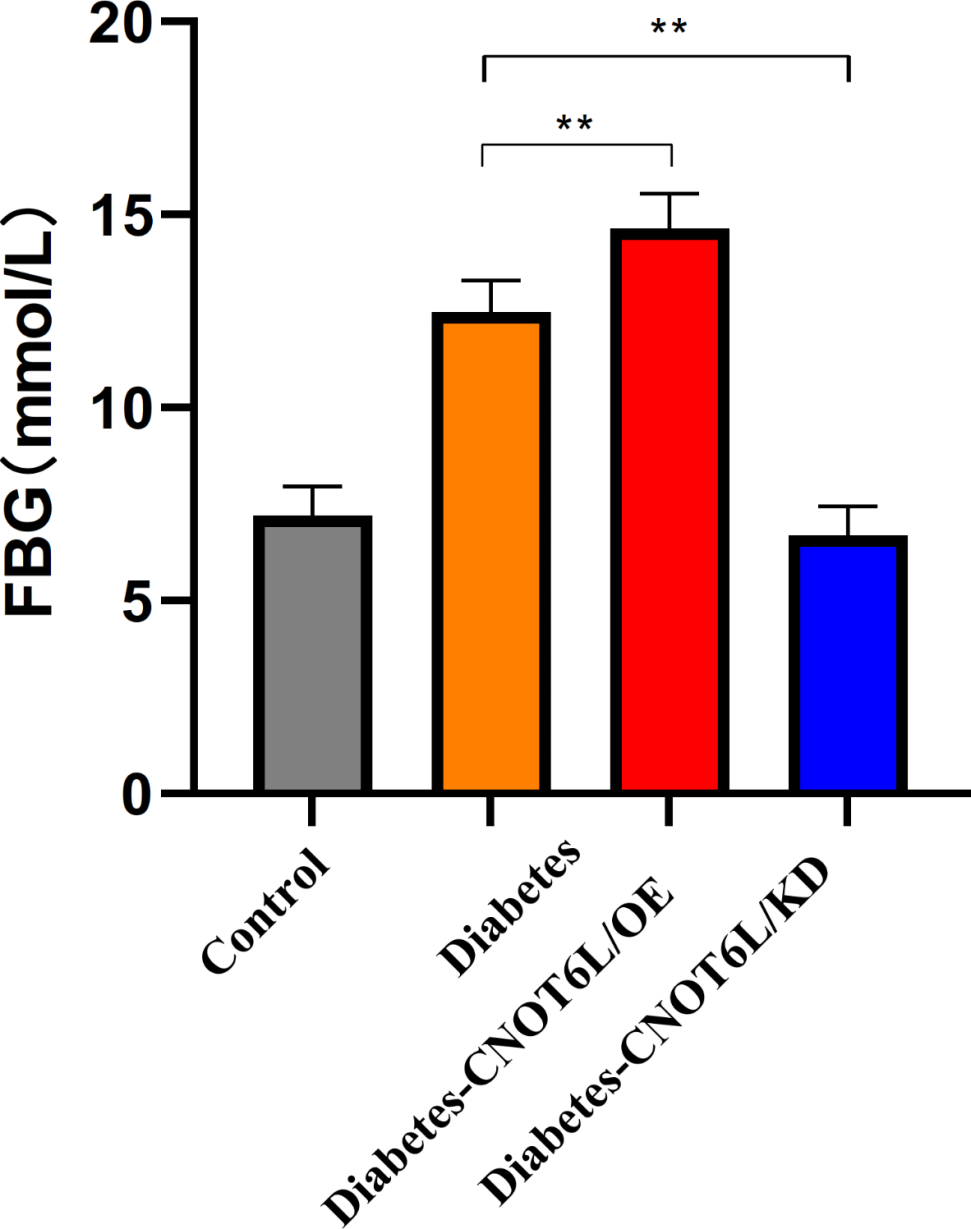
Blood glucose levels in type 2 diabetes mellitus mice. Glucose levels were determined for the control, type 2 diabetes (Diabetes), type 2 diabetes CNOT6L gene overexpression (Diabetes-CNOT6L/OE), and type 2 diabetes CNOT6L gene knockout (Diabetes-CNOT6L/KO) groups. The results are presented as mean ± standard deviation of independent 10 experiments. ** *P* < 0.01.

Blood glucose level measurements in the mouse model showed that the T2D group had significantly higher blood glucose levels than those in the non-disease-afflicted mice (control). In the T2D-CNOT6L/OE group, blood glucose levels were significantly higher than those in the T2D group(*P* < 0.01). In the T2D-CNOT6L/KO group, blood glucose levels were significantly lower than those in the T2D group (*P* < 0.01) (Fig. 16).

## Discussion

T2Dis a chronic metabolic disorder that poses a serious threat to various organs^12^. Patients with T2D are more susceptible to heart disease, hypertension, and stroke, which are leading causes of mortality. Elevated blood glucose levels can impair kidney filtration, leading to diabetic nephropathy, which may necessitate dialysis or transplantation. Furthermore, nerve function can be compromised, resulting in diabetic neuropathy characterized by sensory abnormalities, pain, and motor dysfunction^13,14^. High blood glucose levels also affect the retinas of the eyes, causing diabetic retinopathy, which can result in blindness. Additionally, compromised circulation due to nerve damage can lead to non-healing lower limb ulcers, potentially requiring amputation. The normal function of the immune system is also affected by high blood glucose levels, making patients more susceptible to infections and impairing wound healing^15,16^. Patients often require regular blood glucose monitoring, insulin injections or medication, and strict adherence to dietary and exercise regimens, significantly impacting their quality of life. The findings of this study indicate that CNOT6L is overexpressed in T2D, with a poorer prognosis associated with higher CNOT6L expression. CNOT6L, a protein involved in gene regulation, may play a role in specific molecular mechanisms in T2D^17^, further influencing disease progression and prognosis, providing insights into the molecular mechanisms of T2D, and suggesting potential strategies for developing targeted therapies involving CNOT6L.

CNOT6L, a component of the Ccr4-Not complex, plays a significant regulatory role within cells and is involved in key processes such as RNA degradation and post-transcriptional regulation^18^. CNOT6L is an RNA-binding protein that, along with other proteins in the Ccr4-Not complex, regulates mRNA degradation and post-transcriptional control. This complex binds to mRNA molecules, mediates their degradation, or regulates their stability, thereby affecting gene expression^19,20^. Despite the limited research on CNOT6L in the context of T2D, emerging studies suggest its involvement in metabolic regulation. For example, CNOT6L has been implicated in modulating glucose metabolism and insulin sensitivity by controlling the stability of specific mRNAs, thereby impacting the expression of genes associated with metabolism^21,22^.Katsumura^10^ proposed that CNOT6L inhibits Gdf15 and Fgf21 mRNA stability in the liver, thereby affecting the corresponding serum protein levels. This indicates that CNOT6L influences metabolism-related gene expression through specific gene regulatory mechanisms. The findings of this study align with those of other studies, indicating the potential role of CNOT6L and its related hub genes in T2D^23^.

Che identified DEGs associated with T2Dusing comprehensive bioinformatics and pathway analyses^23^. They found that 20 upregulated genes were enriched in the regulation of mRNA, protein binding, and phospholipase D signaling pathways, whereas 10 downregulated genes were enriched in telomere maintenance through semi-conservative replication, AGE-RAGE signaling pathways in diabetes complications, and insulin resistance pathways. Among these, the core gene identified was CNOT6L.

CNOT6L facilitates the degradation of specific mRNAs related to metabolism, consequently diminishing the expression levels of these genes. This modulation can impact the functionality of metabolic pathways, thereby influencing glucose metabolism and other metabolic processes, consequently significantly affecting the onset and progression of diabetes. Abnormal glucose metabolism stands out as one of the primary features of diabetes^24^. Under normal circumstances, insulin is a crucial hormone that helps cells absorb glucose, thereby maintaining blood glucose levels within the normal range. However, in patients with diabetes, insulin function is impaired and cells become less responsive to glucose, leading to high blood sugar levels^25^.

CNOT6L may regulate the pathogenesis of T2D by affecting hsa-miR-9-5p, which modulates cellular functions by regulating mRNA expression levels. CNOT6L plays a role in RNA degradation complexes by regulating the stability of miRNAs^26,27^, which typically regulate gene expression by interacting with target mRNA molecules^28,29^. Consequently, CNOT6L may impact miRNA function by modulating interactions between hsa-miR-9-5p and specific mRNAs, thus regulating the interaction between hsa-miR-9-5p and crucial molecules within signaling pathways, consequently influencing their activity^30^. Dysregulation of CNOT6L in these processes can directly affect phenotypic outcomes, leading to poor prognosis^31^.

Additionally, this study found that CNOT6L interacts with miRNAs such as hsa-miR-106b-5p, hsa-miR-186-5p, hsa-miR-146a-5p; hsa-miR-17-5p, hsa-miR-18a-5p, hsa-miR-19a-3p, suggesting that CNOT6L may influence the activity of signaling pathways associated with type 2 diabetes by regulating the stability of these miRNAs, among other factors, thereby impacting disease progression.

The results of this study indicate that the core genes (CNOT6L and GRIN2B) are associated with diabetes, T2D, diabetic complications, abnormal blood lipids, high blood sugar, and inflammation. T2D is often accompanied by inflammatory and metabolic abnormalities^32^. CNOT6L may additionally impact the expression of genes associated with inflammation or metabolism through its regulation of hsa-miR-9-5p. Hsa-miR-9-5p is presumed to play a role in modulating specific signaling pathway molecules, such as those involved in blood coagulation or ubiquitination, such as PAI-1 and UBC. This modulation could potentially alter the activity of these pathways, resulting in changes in gene expression that ultimately influence disease progression.

The regulation of hsa-miR-9-5p by CNOT6L may affect cellular physiological processes, such as apoptosis, proliferation, and differentiation. Mittal et al.^33^ explored the relationship between the Ccr4a (CNOT6) and Ccr4b (CNOT6L) adenosine nucleotidase subunits of the human Ccr4-Not complex and cell death/aging. They showed that CNOT6 and CNOT6L regulate different sets of genes. They identified CNOT6L as a critical regulator of insulin-like growth factor-binding protein 5, which mediates cell cycle arrest and aging by modulating p53. These physiological processes may be related to inflammation or metabolism, and the regulatory role of CNOT6L may influence the expression of relevant genes by affecting these processes.

In summary, as a pivotal regulatory protein, CNOT6L likely assumes multiple roles in the pathogenesis of T2D. One crucial function involves its modulation of metabolism-related gene expression by regulating mRNA stability, thereby impacting glucose metabolism and insulin sensitivity, consequently influencing the prognosis of T2D. Additionally, it may exert its influence on T2D by regulating the expression of characteristic genes associated with inflammation. Although this study employed rigorous bioinformatic analysis, further research, including gene overexpression or knockout animal experiments, is imperative to validate its functional role.

## Conclusion

In conclusion, CNOT6L induces hyperglycemia, inflammation and other changes through related miRNA, insulin, PPAR and other related signaling pathways, thus affecting the prognosis of type 2 diabetes.

## Electronic supplementary material

Below is the link to the electronic supplementary material.

Supplementary Material 1

Supplementary Material 2

Supplementary Material 3

Supplementary Material 4

## Data Availability

The datasets used or analysed during the current study are available from the corresponding author on reasonable request.

## Abbreviations

DEGs: Differential epigenetic genes
WGCNA: Weighted gene co-expression network analysis
MAD: Median absolute deviation
PPI: Protein–protein interaction
STRING: Search Tool for the Retrieval of Interacting Genes
GO: Gene ontology
KEGG: Kyoto Encyclopedia of Genes and Genomes
GSEA: Gene set enrichment analysis
CTD: Comparative toxicogenomics database
Diabetes-CNOT6L/OE: T2D CNOT6L gene overexpression group
Diabetes: T2D
Diabetes-CNOT6L/KO: T2D CNOT6L gene knockout group
Diabetes-CNOT6L/KO: T2D CNOT6L gene knockout group
WB: Western blotting
RT-qPCR: Reverse transcription quantitative real-time polymerase chain reaction
MCC: Matthews correlation coefficient
MNC: Maximum neighborhood component
SDS-PAGE: Sodium dodecyl sulfate–polyacrylamide gel electrophoresis
ECL: Enhanced chemiluminescence
PVDF: Polyvinylidene fluoride
ME: Module eigengene
MM: Module membership

## References

[1] Artasensi, A., Pedretti, A., Vistoli, G. & Fumagalli, L. Type 2 diabetes mellitus: A review of multi-target drugs. * Molecules*** 25**(8), 1987 (2020). 10.3390/molecules25081987 PMC7221535 32340373

[2] Tinajero, M. G. & Malik, V. S. An update on the epidemiology of type 2 diabetes: A global perspective. *Endocrinol. Metab. Clin. North. Am.*** 50** (3), 337–355 (2021). 34399949 10.1016/j.ecl.2021.05.013

[3] Yan, Y. et al. Prevalence, awareness and control of type 2 diabetes mellitus and risk factors in Chinese elderly population. *BMC Public. Health*. **22** (1), 1382 (2022). 35854279 10.1186/s12889-022-13759-9 PMC9295461

[4] Landgraf, R. et al. Therapy of type 2 diabetes. *Exp. Clin. Endocrinol. Diabetes*. **127**, 01 (2019). 10.1055/a-1018-9106 31860927

[5] Damanik, J. & Yunir, E. Type 2 diabetes mellitus and cognitive impairment. *Acta Med. Indones*. **53** (2), 213–220 (2021). 34251351

[6] da Rocha, R. B., Silva, C. S. & Cardoso, V. S. Self-care in adults with type 2 diabetes mellitus: A systematic review. *Curr. Diabetes Rev.*** 16** (6), 598–607 (2020). 31267873 10.2174/1573399815666190702161849

[7] Ma, Q. et al. Research progress in the relationship between type 2 diabetes mellitus and intestinal flora. *Biomed. Pharmacother*. **117**, 109138 (2019). 31247468 10.1016/j.biopha.2019.109138

[8] Chen, C., Hou, J., Tanner, J. J. & Cheng, J. Bioinformatics methods for mass spectrometry-based proteomics data analysis. *Int. J. Mol. Sci.*** 21** (8), 2873 (2020). 32326049 10.3390/ijms21082873 PMC7216093

[9] Fu, Y., Ling, Z., Arabnia, H. & Deng, Y. Current trend and development in bioinformatics research. *BMC Bioinform.*** 21** (Suppl 9), 538 (2020). 10.1186/s12859-020-03874-y PMC7713158 33272214

[10] Tysoe, O. CNOT6L regulates hepatokine expression. *Nat. Rev. Endocrinol.*** 18** (7), 392 (2022). 35523890 10.1038/s41574-022-00684-5

[11] Laakso, M. Biomarkers for type 2 diabetes. *Mol. Metab.*** 27S** (Suppl), S139–S146 (2019). 31500825 10.1016/j.molmet.2019.06.016 PMC6768493

[12] Gloyn, A. L. & Drucker, D. J. Precision medicine in the management of type 2 diabetes. *Lancet Diabetes Endocrinol.*** 6** (11), 891–900 (2018). 29699867 10.1016/S2213-8587(18)30052-4

[13] Srikanth, V., Sinclair, A. J., Hill-Briggs, F., Moran, C. & Biessels, G. J. Type 2 diabetes and cognitive dysfunction-towards effective management of both comorbidities. *Lancet Diabetes Endocrinol.*** 8** (6), 535–545 (2020). 32445740 10.1016/S2213-8587(20)30118-2

[14] Peer, N., Balakrishna, Y. & Durao, S. Screening for type 2 diabetes mellitus. *Cochrane Database Syst. Rev.*** 5** (5), CD005266 (2020). 32470201 10.1002/14651858.CD005266.pub2 PMC7259754

[15] Jing, X. et al. Related factors of quality of life of type 2 diabetes patients: A systematic review and meta-analysis. *Health Qual. Life Outcomes*. **16** (1), 189 (2018). 30231882 10.1186/s12955-018-1021-9 PMC6147036

[16] Taylor, R., Ramachandran, A., Yancy, W. S. Jr & Forouhi, N. G. Nutritional basis of type 2 diabetes remission. *BMJ*. **374**, n1449 (2021). 34233884 10.1136/bmj.n1449 PMC8261662

[17] Khosla, S., Samakkarnthai, P., Monroe, D. G. & Farr, J. N. Update on the pathogenesis and treatment of skeletal fragility in type 2 diabetes mellitus. *Nat. Rev. Endocrinol.*** 17** (11), 685–697 (2021). 34518671 10.1038/s41574-021-00555-5 PMC8605611

[18] Sha, Q. Q. et al. CNOT6L couples the selective degradation of maternal transcripts to meiotic cell cycle progression in mouse oocyte. *EMBO J.*** 37** (24), e99333 (2018). 30478191 10.15252/embj.201899333 PMC6293276

[19] Chioccarelli, T. et al. FUS driven circCNOT6L biogenesis in mouse and human spermatozoa supports zygote development. *Cell. Mol. Life Sci.*** 79** (1), 50 (2021). 34936029 10.1007/s00018-021-04054-8 PMC8739325

[20] Dai, X. X. et al. CNOT6/6L-mediated mRNA degradation in ovarian granulosa cells is a key mechanism of gonadotropin-triggered follicle development. *Cell. Rep.*** 37** (7), 110007 (2021). 34788619 10.1016/j.celrep.2021.110007

[21] Li, C. Y. et al. Cytidine-containing tails robustly enhance and prolong protein production of synthetic mRNA in cell and in vivo. *Mol. Ther. Nucleic Acids*. **30**, 300–310 (2022). 36320322 10.1016/j.omtn.2022.10.003 PMC9614650

[22] Jiang, Z. Y. & Fan, H. Y. Five questions toward mRNA degradation in oocytes and preimplantation embryos: when, who, to whom, how, and why?†. *Biol. Reprod.*** 107** (1), 62–75 (2022). 35098307 10.1093/biolre/ioac014

[23] Che, X. et al. Differently expressed genes (DEGs) relevant to type 2 diabetes mellitus identification and pathway analysis via integrated bioinformatics analysis. *Med. Sci. Monit.*** 25**, 9237–9244 (2019). 31797865 10.12659/MSM.918407 PMC6909911

[24] Chen, J. et al. Gastrointestinal consequences of type 2 diabetes mellitus and impaired glycemic homeostasis: A mendelian randomization study. *Diabetes Care*. **46** (4), 828–835 (2023). 36800530 10.2337/dc22-1385 PMC10091506

[25] Rachdaoui, N. Insulin: The friend and the foe in the development of type 2 diabetes mellitus. *Int. J. Mol. Sci.*** 21** (5), 1770 (2020). 32150819 10.3390/ijms21051770 PMC7084909

[26] Wang, H., Radomska, H. S. & Phelps, M. A. Replication study: Coding-independent regulation of the tumor suppressor PTEN by competing endogenous mRNAs. *Elife*. **9**, e56651 (2020). 33073769 10.7554/eLife.56651 PMC7572125

[27] Morita, M. et al. Hepatic posttranscriptional network comprised of CCR4-NOT deadenylase and FGF21 maintains systemic metabolic homeostasis. *Proc. Natl. Acad. Sci. U S A*. **116** (16), 7973–7981 (2019). 30926667 10.1073/pnas.1816023116 PMC6475422

[28] Correia de Sousa, M., Gjorgjieva, M., Dolicka, D., Sobolewski, C. & Foti, M. Deciphering miRNAs’ action through miRNA editing. *Int. J. Mol. Sci.*** 20** (24), 6249 (2019). 31835747 10.3390/ijms20246249 PMC6941098

[29] Chen, L. et al. Trends in the development of miRNA bioinformatics tools. *Brief. Bioinform*. **20** (5), 1836–1852 (2019). 29982332 10.1093/bib/bby054 PMC7414524

[30] Bukas, C. et al. Echo2Pheno: A deep-learning application to uncover echocardiographic phenotypes in conscious mice. *Mamm. Genome*. **34** (2), 200–215 (2023). 37221250 10.1007/s00335-023-09996-x PMC10290584

[31] Eskandarian, S., Grand, R., Irani, S., Saeedi, M. & Mirfakhraie, R. Importance of CNOT8 deadenylase subunit in DNA damage responses following ionizing radiation (IR). *Rep. Biochem. Mol. Biol.*** 9** (2), 163–170 (2020). 33178865 10.29252/rbmb.9.2.163 PMC7603255

[32] Shan, Z., Fa, W. H., Tian, C. R., Yuan, C. S. & Jie, N. Mitophagy and mitochondrial dynamics in type 2 diabetes mellitus treatment. *Aging (Albany NY)*. **14** (6), 2902–2919 (2022). 35332108 10.18632/aging.203969 PMC9004550

[33] Mittal, S., Aslam, A., Doidge, R., Medica, R. & Winkler, G. S. The Ccr4a (CNOT6) and Ccr4b (CNOT6L) deadenylase subunits of the human Ccr4-Not complex contribute to the prevention of cell death and senescence. *Mol. Biol. Cell.*** 22** (6), 748–758 (2011). 21233283 10.1091/mbc.E10-11-0898 PMC3057700

