# Author Correction: High expression of CNOT6L contributes to the negative development of type 2 diabetes

**DOI:** 10.1038/s41598-024-83685-w

**Published:** 2024-12-23

**Authors:** Yuna Zhang, Guihong Liu, Haiyan Ding, Bingge Fan

**Affiliations:** Department of Endocrinology, The Fourth Hospital of Hebei Medical University, Shijiazhuang, 050011 China

Correction to: *Scientific Reports* 10.1038/s41598-024-76095-5, published online 21 October 2024

In the original version of this Article Affiliation 1 was incorrectly given as ‘Department of Endocrinology, The Forth Hospital of Hebei Medical University, Shijiazhuang, 050011, China’. The correct affiliation is listed below.

Department of Endocrinology, The Fourth Hospital of Hebei Medical University, Shijiazhuang, 050011, China

The original Article has been corrected.

