# Supplementary material for: High expression of CNOT6L contributes to the negative development of type 2 diabetes
Source: Sci Rep. 2024 Oct 21;14:24723. doi: 10.1038/s41598-024-76095-5 (PMC11494123; doi:10.1038/s41598-024-76095-5)
Supplement: Supplementary file 1 — Supplementary Material 1 [file 41598_2024_76095_MOESM1_ESM.pdf]

KEGG Database Project
Kanehisa Laboratories
www.kanehisa.jp

Ref: 240290

Permission is granted to Scientific Reports of Springer Nature Ltd to publish both in print and digital under the CC BY 4.0 open access license the following KEGG pathway map image in the article "High expression of CNOT6L contributes to the negative development of type 2 diabetes" written by Fan Bingge and colleagues:

- PPAR signaling pathway (map03320)

subject to the condition that the original source is acknowledged by citing at least one KEGG paper.

Permission granted: Naomi Takahashi

Naomi Takahashi, Kanehisa Laboratories

Date: 3 April 2024

Copyright holder: Kanehisa Laboratories
