# Supplementary material for: High expression of CNOT6L contributes to the negative development of type 2 diabetes
Source: Sci Rep. 2024 Oct 21;14:24723. doi: 10.1038/s41598-024-76095-5 (PMC11494123; doi:10.1038/s41598-024-76095-5)
Supplement: Supplementary file 2 — Supplementary Material 2 [file 41598_2024_76095_MOESM2_ESM.docx]

**2 Methods**

**2.3 Experimental Approach**

Plasmids were constructed using the gene knockout and over expressed sequences. The gene knockout sequence (TableS1) and overexpression sequence are as follows:

**TableS1 Gene knockout sequence**

| **GENE** | **Species** | **Position** | **SS Sequence (5' -> 3')** | **SS Sequence (5' -> 3')** |
| --- | --- | --- | --- | --- |
| CONT6L | Mus musculus | 394 | GGUGUUGCCUUAUGAACUUTT | AAGUUCAUAAGGCAACACCTT |
| CONT6L | Mus musculus | 427 | GCUACAAACUCUAGGUUUATT | UAAACCUAGAGUUUGUAGCTT |
| CONT6L | Mus musculus | 958 | CCAGGUAGCAAUGGCAAAUTT | AUUUGCCAUUGCUACCUGGTT |
| CONT6L | Mus musculus | 1301 | GGUGUUGUGGAAUAUUUAATT | UUAAAUAUUCCACAACACCTT |

The gene over expression sequence was as follows:

>NM_001285511.1:197-1849 *Mus musculus* Ccr4-Not transcription complex, subunit 6-like (Cnot6l), transcriptvariant3, mRNA ATGCCAAAGGAAAAATATGATCCTCCAGATCCTCGCAGAATTTACACCATCATGTCCGCAGAGGAGGTAGCCAATGGGAAAAAATCTCACTGGGCAGAGTTAGAGATCTCGGGTAGAGTGCGGAGCTTAAGTACATCACTCTGGTCATTAACACACTTGACAGCACTGCACCTAAATGACAATAACCTTGCTCGCATTCCACCTGATATTGCCAAGCTTCATAATCTGGTTTACCTGGATCTGTCATCCAATAAACTCAGAAGTTTACCAGCAGAACTAGGAAACATGGTGTCTCTCAGGGAATTGCTTTTAAATGACAATTATTTACGGGTGTTGCCTTATGAACTTGGCCGGCTCTTCCAGCTACAAACTCTAGGTTTAACAGGCAATCCTTTATCACAGGATATTATGAGCTTATACCAGGACCCAGATGGAACCCGAAAGCTACTGAACTTCATGCTTGACAATCTTGCAGTTCATCCAGAGCAGCTTCCTCCGAGGCCATGGATTACATTAAAAGAACGAGACCAAATTCTGCCATCAGCATCATTCACGGTTATGTGTTACAATGTGTTATGTGATAAATATGCTACCAGGCAGCTATATGGCTATTGTCCGTCCTGGGCATTAAACTGGGAATACAGGAAAAAGGGAATTATGGAAGAAATTGTTAACTGGGACGCAGATATCATTAGTCTTCAGGAAGTGGAAACAGAGCAATACTTTACTCTCTTTCTGCCAGCATTGAAGGATCGTGGATATGATGGATTTTTTTCTCCAAAGTCACGTGCCAAAATCATGTCTGAGCAGGAAAGAAAGCATGTGGATGGTTGTGCAATATTCTTCAAAACAGAAAAATTTACATTGGTGCAGAAGCATACAGTGGAATTCAACCAGGTAGCAATGGCAAATTCAGATGGATCCGAAGCAATGCTAAACAGAGTAATGACGAAAGATAACATTGGCGTTGCTGTGGTGTTAGAGGTCCACAAGGAGCTTTTTGGAACAGGTATGAAGCCTATTCATGCTGCAGACAAACAGCTGCTTATAGTGGCAAATGCCCACATGCATTGGGACCCAGAGTATTCTGATGTGAAACTTATTCAGACCATGATGTTTGTCTCAGAGGTTAAAAACATTCTGGAGAAAGCCTCAAGTAGGCCTGGCAGCCCAACTGCAGATCCCAATTCCATCCCGCTGGTGCTATGTGCAGATCTTAACTCATTGCCAGATTCAGGTGTTGTGGAATATTTAAGCAACGGTGGAGTAGCTGACAACCATAAAGACTTCAAGGAACTAAGGTACAATGAGTGTCTTATGAACTTCAGCTGTAGTGGAAAGAATGGAAGCTCAGAAGGGAGAATCACACATGGCTTCCAACTTAAGAGCGCCTATGAAAATAACTTGATGCCTTATACCAATTACACCTTTGATTTCAAAGGTGTGATTGACTACATTTTCTATTCCAAGACTCATATGAACGTGCTTGGTGTCCTGGGGCCTTTAGATCCTCAATGGCTGGTTGAGAACAACATCACTGGGTGTCCACACCCTCACATCCCTTCAGACCACTTCTCACTGTTAACACAACTTGAACTCCACCCTCCACTCCTGCCTCTTGTCAATGGTGTTCACTTGCCTAATCGGAGGTAG

**2.3.1 Western Blotting (WB)**

Total protein extraction: One volume of fresh mouse whole blood was mixed with three volumes of red blood cell lysis buffer (R1010; Solarbio, Beijing, China) and thoroughly mixed by gently inverting the tubes. The mixture was left on ice for 15 min and gentle inversion was performed twice. The cells were collected by centrifugation at 4°C and 450×*g* for 10 min to pellet the cells, and the supernatant was carefully removed. Red blood cell lysis buffer equal to twice the volume of whole blood was added to the cell pellet, and the cells were gently resuspended by shaking. The mixture was then placed in a centrifuge (5810R; Eppendorf, Germany) and centrifuged at 4°C and 450×*g* for 10 min to pellet the cells, and the supernatant was again carefully and thoroughly aspirated. The cells were resuspended in RIPA lysis buffer (G2002; Servicebio, Wuhan, China) containing protease inhibitors, placed on ice, and lysed for 30 min. After lysis, the samples were centrifuged at 12,000×*g* for 10 min and the supernatant, which represented the total protein solution, was collected.

Protein concentration determination: Proteins were quantified using the BCA protein assay and a standard curve was generated using a spectrophotometer (MK3; Thermo Fisher Scientific).

Protein denaturation: Add the protein solution to the 5× reducing protein loading buffer at a ratio of 4:1, and subject it to denaturation in a boiling water bath for 15 minutes. Then, store the denatured proteins in a -20°C freezer for later use.

Electrophoresis and film transfer: Proteins were separated by sodium dodecyl sulfate-polyacrylamide gel electrophoresis (SDS-PAGE) and subsequently transferred onto a polyvinylidene fluoride (PVDF) membrane. The membrane was then blocked with 5% BSA at room temperature for 1 h. Anti-CNOT6L (dilution 1:1000, ab103659; Abcam, Shanghai, China), anti-PPARγ (dilution 1:5000, 66936-1-Ig; Proteintech, Wuhan, China), and anti-GAPDH (dilution 1:50000, 60004-1-Ig; Proteintech) were used as primary antibodies for detection. GAPDH was used as the internal control. The membrane was incubated overnight with the primary antibodies on a shaker at 4°C. After the overnight incubation, the primary antibodies were removed, and the membrane was washed three times with TBST for 5–10 min, and the incubated for 1 h at room temperature with the appropriate species-specific secondary antibodies (dilution 1:5000). The PVDF membrane was removed, placed on absorbent paper to remove excess liquid, and then placed on the tray of a chemiluminescence imager. Enhanced Chemiluminescence (ECL) luminescent liquid was added to ensure that the membrane was fully immersed in the liquid. After 1 min of incubation, the membrane was placed in a chemiluminescence imager and the chemiluminescence reaction was initiated according to the preset program. The data were saved and subsequent data analysis was performed using AIWBwell™.

**2.3.2 Real-time polymerase chain reaction (RT-qPCR)**

The RT-qPCR experimental setup was identical to that used for WB. The steps of RT-qPCR are as follows:

Total RNA Extraction: Take 1ml of whole blood and centrifuge it at 3000rpm for 5 minutes using a centrifuge (Eppendorf, 5810R, Germany), discard the supernatant. Then add 3mL of red blood cell lysis buffer, vigorously vortex to mix, and let it stand at room temperature for 10 minutes. Centrifuge again under the same conditions, discard the supernatant. Repeat the red blood cell lysis buffer treatment 1-2 times until the liquid is clear. Centrifuge to collect the precipitate, add 1ml of RNA extraction reagent, and vortex to mix. Phase separation: After pretreatment of the sample, centrifuge at 12000rpm for 10 minutes at 4°C, transfer the supernatant to a new centrifuge tube. Add 100 μl of chloroform substitute, invert the centrifuge tube for 15 seconds to mix thoroughly, let it stand for 3 minutes, and centrifuge at 12000rpm for 10 minutes at 4°C. Transfer 400 μl of the supernatant to a new centrifuge tube. RNA precipitation: Add 550 μl of isopropanol, invert to mix thoroughly. Incubate at -20°C for 15 minutes. Then centrifuge at 12000rpm for 10 minutes at 4°C, and the white precipitate at the bottom of the tube is RNA. RNA washing: Remove the liquid, add 1ml of 75% ethanol, invert to mix and wash the precipitate. Centrifuge at 12000rpm for 5 minutes at 4°C, remove the liquid completely. RNA dissolution: Place the centrifuge tube on a clean bench and blow for 3-5 minutes, add 15μl of RNA dissolution solution to dissolve the RNA, and incubate at 60°C for 10 minutes. RNA concentration determination: Use a NanoDrop2000 ultramicro spectrophotometer (Thermo) to measure the concentration and purity of RNA: After zeroing the instrument blank, take 2.5μl of the test RNA solution on the detection pedestal, lower the sample arm, and start the absorbance measurement using the software on the computer.

Reverse Transcription: According to a 20 μL reaction system, 4 μL of 5×SweScript All-in-One SuperMix for qPCR, 1 μL of gDNA Remover, 2 μg of Total RNA, and RNase-free water were added to a final volume of 20 μL. The mixture was gently mixed and centrifuged, and reverse transcription was performed.

Quantitative PCR: Take 0.1ml of the PCR reaction plate and set up the reaction system as follows: 7.5 µL of 2×Universal Blue SYBR Green qPCR Master Mix(ES Science，QP002, ShangHai, China); 1.5 µL of F/R Primers (2.5μM); 2.0 µL of cDNA; and 4.0 µL of Nuclease-Free Water. Each sample was run in triplicate. Seal the plate using an automated sealer and centrifuge it using a microplate centrifuge. Set up the PCR amplification program (StepOneTM) as follows: firstly, denaturation at 95°C for 30s; followed by 40 cycles of denaturation at 95°C for 15s and annealing/extension at 60°C for 30s; finally, perform a melting curve analysis by heating from 65°C to 95°C with an increment of 0.5°C per step, collecting fluorescence signals at each step. Analyze results using the 2-ΔΔCT method.

The primer sequences (5′–3′) for the target genes were as follows (Table S2):

Table S2 The primer sequences (5′–3′) for the target genes

| Gene | Direction | Sequence (5' -> 3') | Length | Tm | Location |
| --- | --- | --- | --- | --- | --- |
| CNOT6L | Forward Primer | AGAAATCTCGGGTAGAGTGCG | 21 | 61.3 | 117-137 |
|  | Reverse Primer | AGCTTGGCAATATCAGGTGGA | 21 | 61.2 | 233-213 |
| GAPDH | Forward Primer | GACCTGCCGTCTAGAAAAACCTGC | 24 | 61.3 | 108-131 |
|  | Reverse Primer | TCGCTGTTGAAGTCAGAGGAGACC | 24 | 62.7 | 108-131 |

**3 Results**

**3.7 Protein and blood glucose levels in mice with type 2 diabetes**

**3.7.1 CNOT6L protein expression（Western blot image of complete membrane length）**


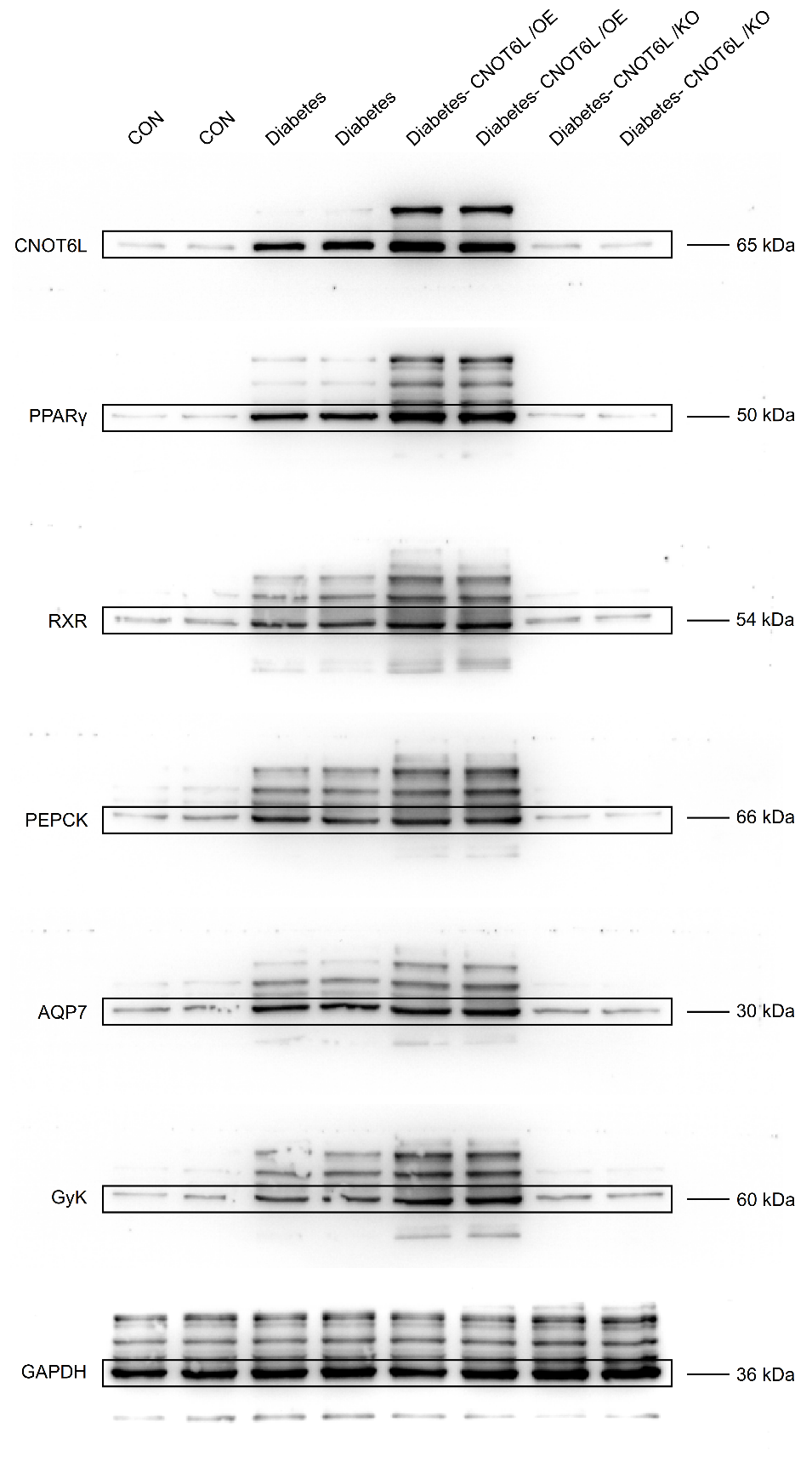


**Figure S1.** Expression of CNOT6L, PPARγ, RXR, PEPCK, AQP7, and GYK in the blood of type 2 diabetes mellitus mice. Protein expression levels were determined by western blotting. A representative blot comparing control (CON), type 2 diabetes (Diabetes), type 2 diabetes CNOT6L gene overexpression (Diabetes-CNOT6L/OE),and type 2 diabetes CNOT6L gene knockout (Diabetes-CNOT6L/KO) groups is shown, with each sample run in duplicate. GAPDH was used as the internal control.


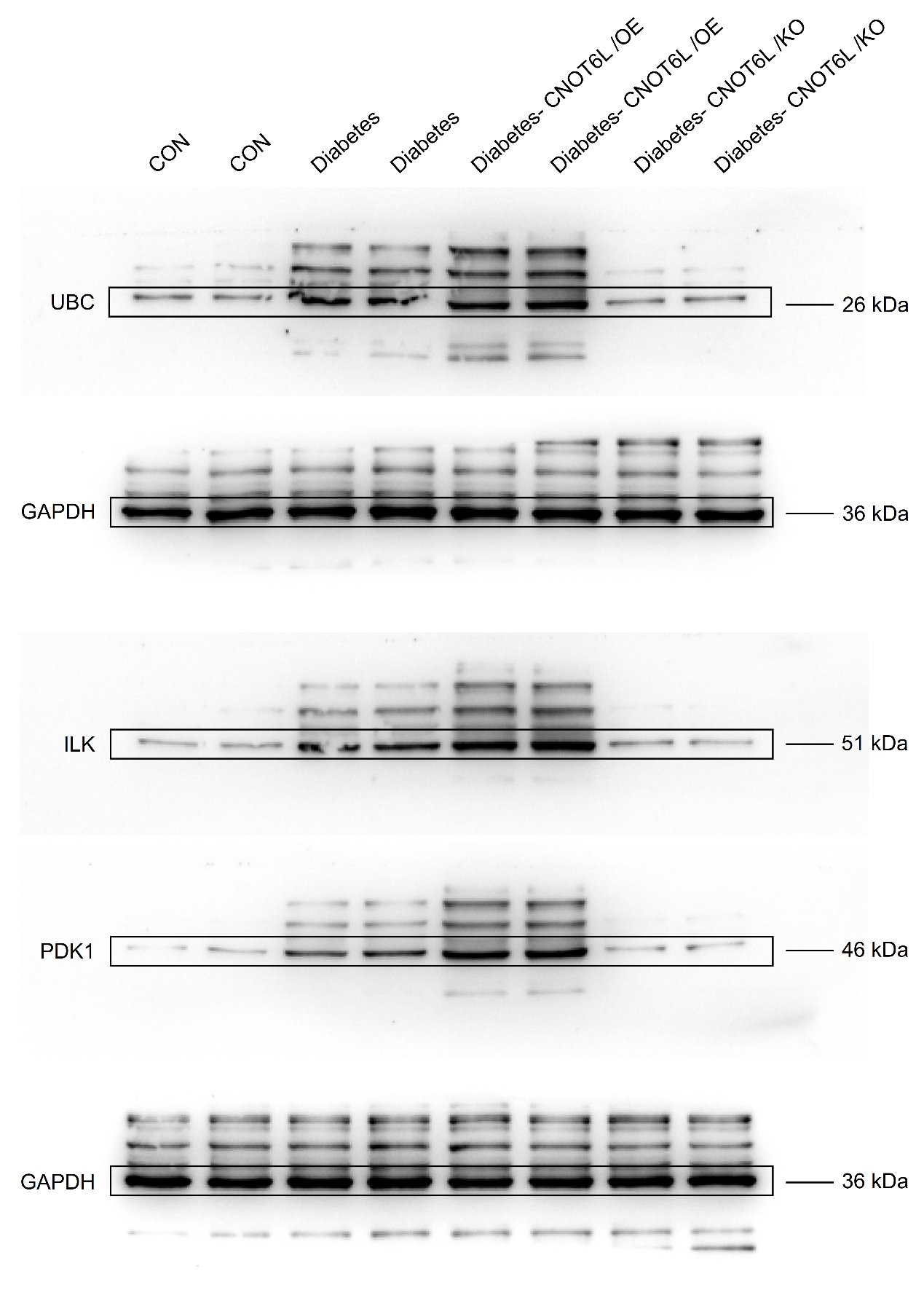


**Figure S2.** Expression of ubiquitination-related genes UBC, ILK, and PDK1 in the blood of type 2 diabetes mellitus mice. Protein expression levels were determined by western blotting. A representative blot comparing control (CON), type 2 diabetes (Diabetes), type 2 diabetes CNOT6L gene overexpression (Diabetes-CNOT6L/OE), and type 2 diabetes CNOT6L gene knockout (Diabetes-CNOT6L/KO) groups is shown, with each sample run in duplicate. GAPDH was used as the internal control.


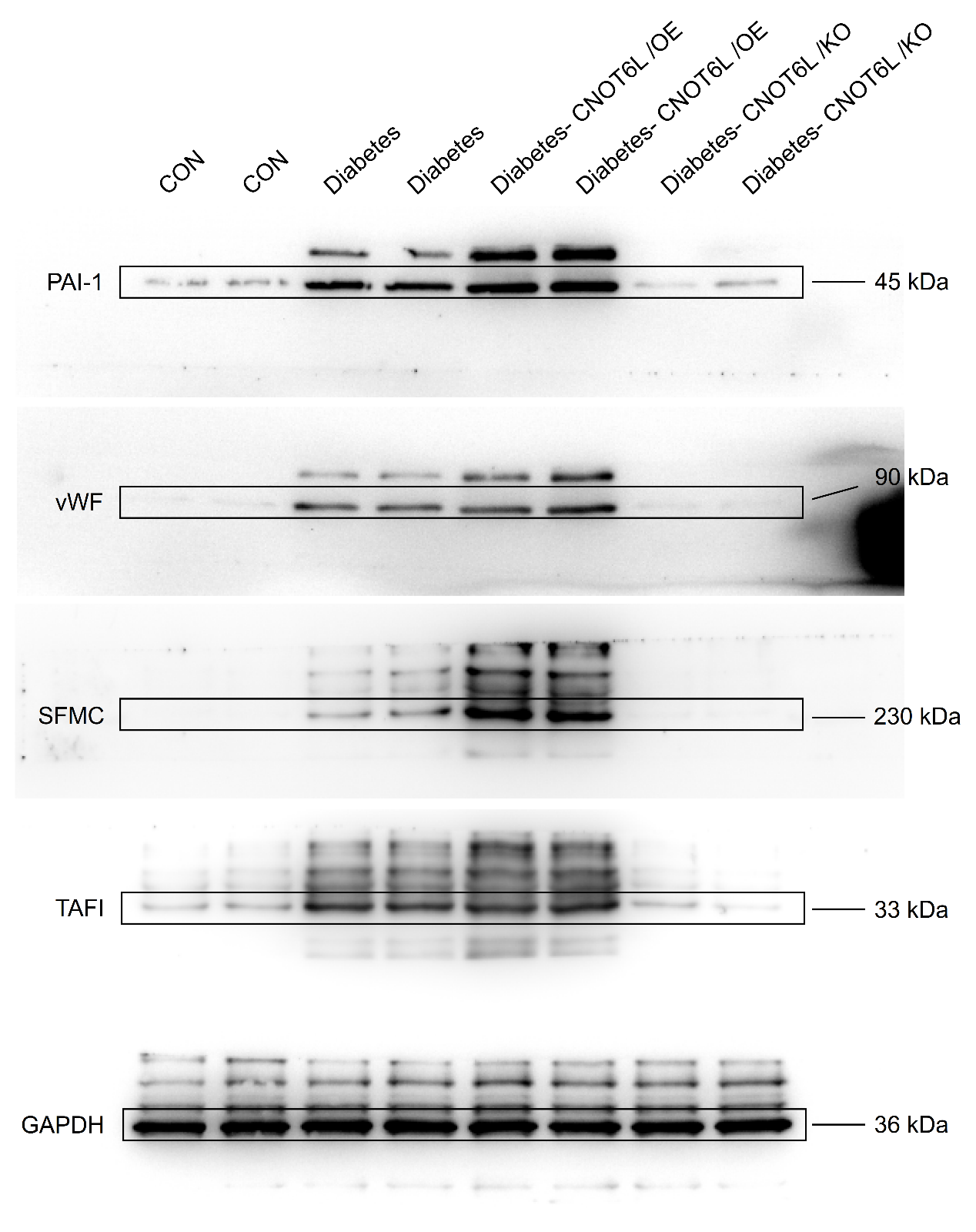


**Figure S3.** Expression of clotting-related genes PAI-1, vWF, SFMC, and TAFI in the blood of type 2 diabetes mellitus mice. Protein expression levels were determined by western blotting. A representative blot comparing control (CON), type 2 diabetes (Diabetes), type 2 diabetes CNOT6L gene overexpression (Diabetes-CNOT6L/OE), and type 2 diabetes CNOT6L gene knockout (Diabetes-CNOT6L/KO) groups is shown, with each sample run in duplicate. GAPDH was used as the internal control.


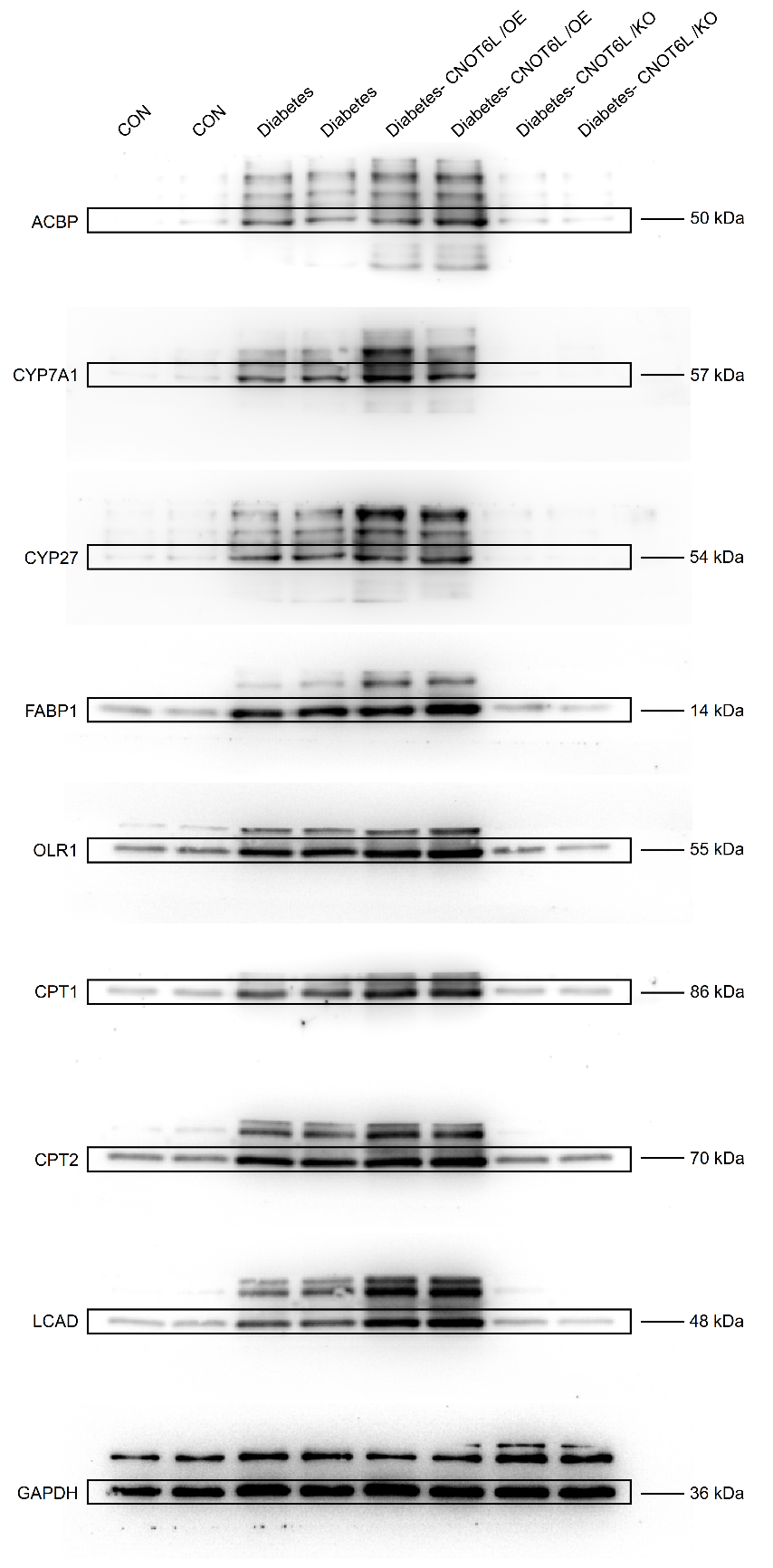


**Figure S4.** Expression of clotting-related genes ACBP, CYP7A1, CYP27, FABP1, OLR1, CPT1, CPT2, and LCAD in the blood of type 2 diabetes mellitus mice. Protein expression levels were determined by western blotting. A representative blot comparing control (CON), type 2 diabetes (Diabetes), type 2 diabetes CNOT6L gene overexpression (Diabetes-CNOT6L/OE), and type 2 diabetes CNOT6L gene knockout (Diabetes-CNOT6L/KO) groups is shown, with each sample run in duplicate. GAPDH was used as the internal control.


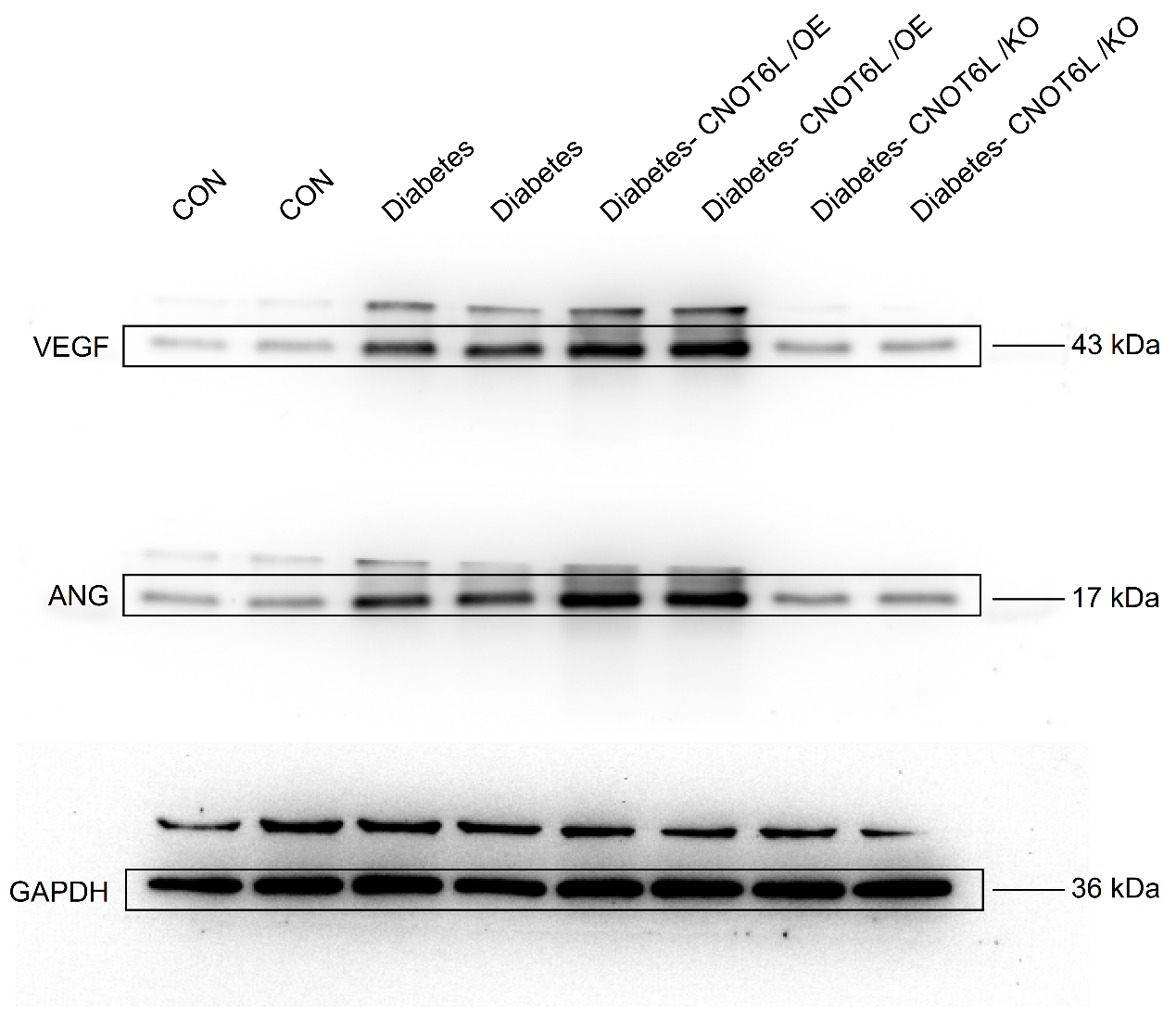


**Figure S5.** Expression of VEGF and ANG in the blood of type 2 diabetes mellitus mice. Protein expression levels were determined by western blotting. A representative blot comparing control (CON), type 2 diabetes (Diabetes), type 2 diabetes CNOT6L gene overexpression (Diabetes-CNOT6L/OE), and type 2 diabetes CNOT6L gene knockout (Diabetes-CNOT6L/KO) groups is shown, with each sample run in duplicate. GAPDH was used as the internal control.


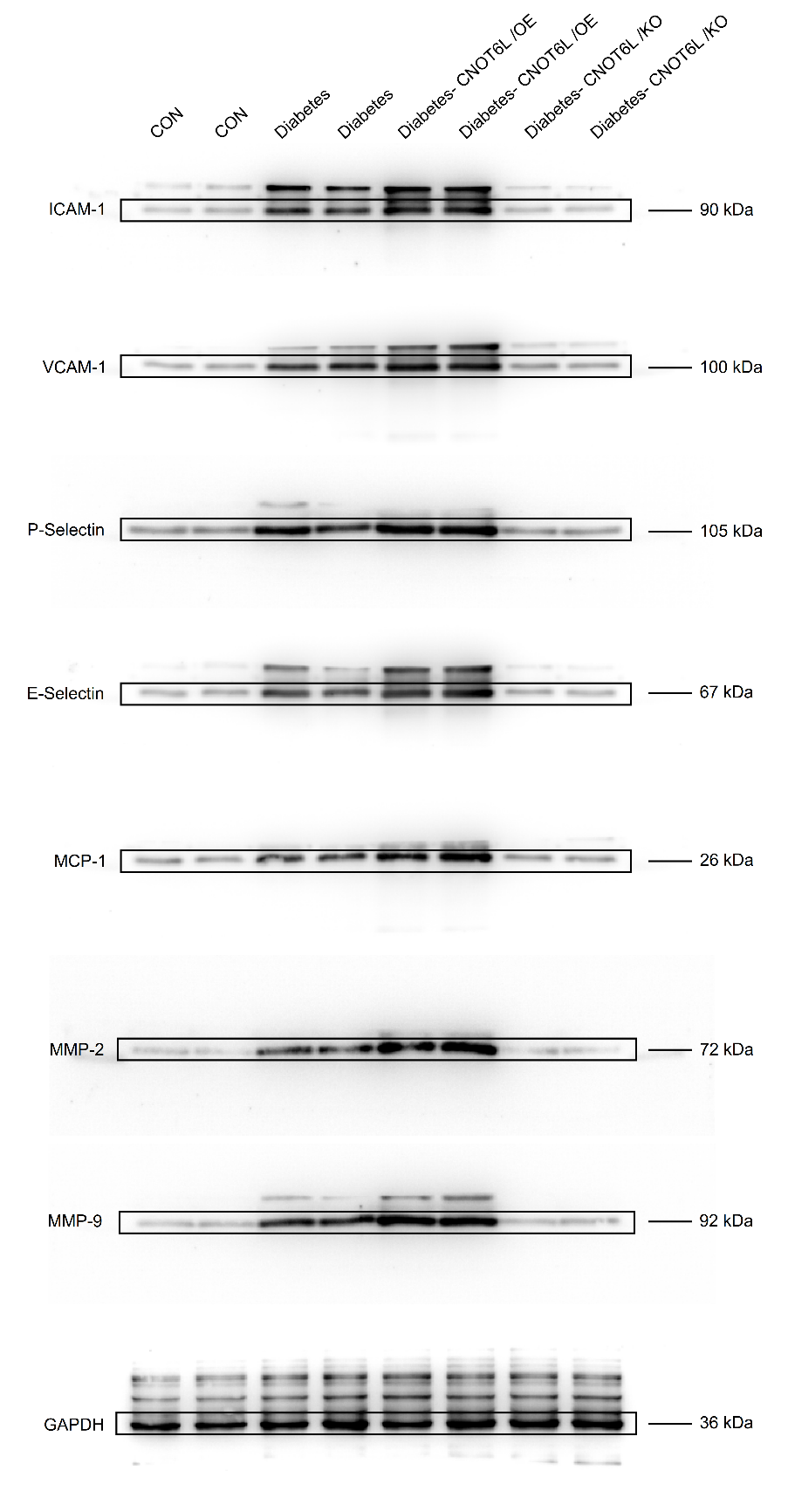


**Figure S6.** Expression of ICAM-1, VCAM-1, P-selectin, E-selectin, MCP-1, MMP-2, and MMP-9 in the blood of type 2 diabetes mellitus mice. Protein expression levels were determined by western blotting. A representative blot comparing control (CON), type 2 diabetes (Diabetes), type 2 diabetes CNOT6L gene overexpression (Diabetes-CNOT6L/OE), and type 2 diabetes CNOT6L gene knockout (Diabetes-CNOT6L/KO) groups is shown, with each sample run in duplicate. GAPDH was used as the internal control.
